# Supplementary material for: Mycobacterial resistance to zinc poisoning requires assembly of P-ATPase-containing membrane metal efflux platforms
Source: Nat Commun. 2022 Aug 12;13:4731. doi: 10.1038/s41467-022-32085-7 (PMC9374683; doi:10.1038/s41467-022-32085-7)
Supplement: Supplementary file 1 — Supplementary information [file 41467_2022_32085_MOESM1_ESM.pdf]

Supplementary information

Supplementary figures 1 to 14

Supplementary tables 1 to 4

Supplementary references

a

```
 PacL1  .......... .......... .......... .......MAI QVFLAKATTT
GroEL1  AKAAVEEGIV PGGGASLIHQ ARKALTELRA SLTGDEVLGV DVFSEALAAP
        401                                                450

 PacL1  VI.....TGL AGVTAYEILK KAAAKAPLRQ TAVSAAALGL RGTRKAEEAA
GroEL1  LFWIAANAGL DGSVVVNKVS ELPAGHGLNV NTLSYGDLAA DGVIDPVKVT
        451                                                500

 PacL1  ESARLKVADV MAEARERIGE ESPTPAISDL HDHDH....
GroEL1  RSAVLNASSV ARMVLTTETV VVDKPAKAED HDHHHGHAH
        501                                   539
```

b

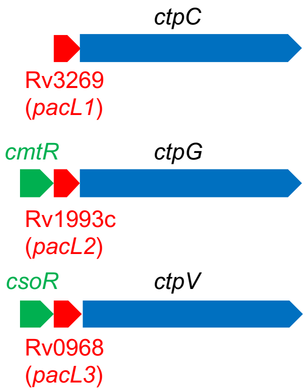

c

```
PacL1 MAIQVFLAKATTTVITGLAGVTAYEILKKAAAKAPLRQTAVSAAALGLRG
PacL2 VVTHELLVKAAGAVLTGLVGVSAYETLRKALGTAPIRRASVTVMEWGLRG
PacL3 MVWHGFLAKAVPTVVTGAVGVAAYEALRKMVVKAPLRAATVSVAAWGIRL
      :. : :*.**. :*:** .**:*** *:*   .**:* ::*:.   *:*

PacL1 TRKAE----EAAESARLKVADVMAEARERIGEESPTPAISDLHD-HDH
PacL2 TRRAE----AAAESARLTVADVVAEARGRIGEEAPLPAGARVDE----
PacL3 AREAERKAGESAEQARLMFADVLAEASERAGEEVPPLAVAGSDDGHDH
      :*.**     :**.*** .***:***  * *** *  * :  .:
```

Supplementary Figure 1. DUF1490-containing proteins in *M. tuberculosis*.

(**a**) Alignment of PacL1 and *M. tuberculosis* GroEL1. Sequences were retrieved from Tuberculist[1] and alignment was performed using MultAlin[2]. Red residues indicate conserved residues.

(**b**) Genetic organization of the *pacL1-ctpC*, *pacL2-ctpG* and *pacL3-ctpV* modules. *cmtR* and *csoR* encode metal-responsive transcriptional regulators[3,4].

(**c**) Sequence alignment of PacL1, PacL2 and PacL3. Sequences were retrieved from Tuberculist[1] and aligned using T-Coffee[5]. Blue residues indicate putative transmembrane domain. Red residues indicate conserved Glu/Ala repeats.

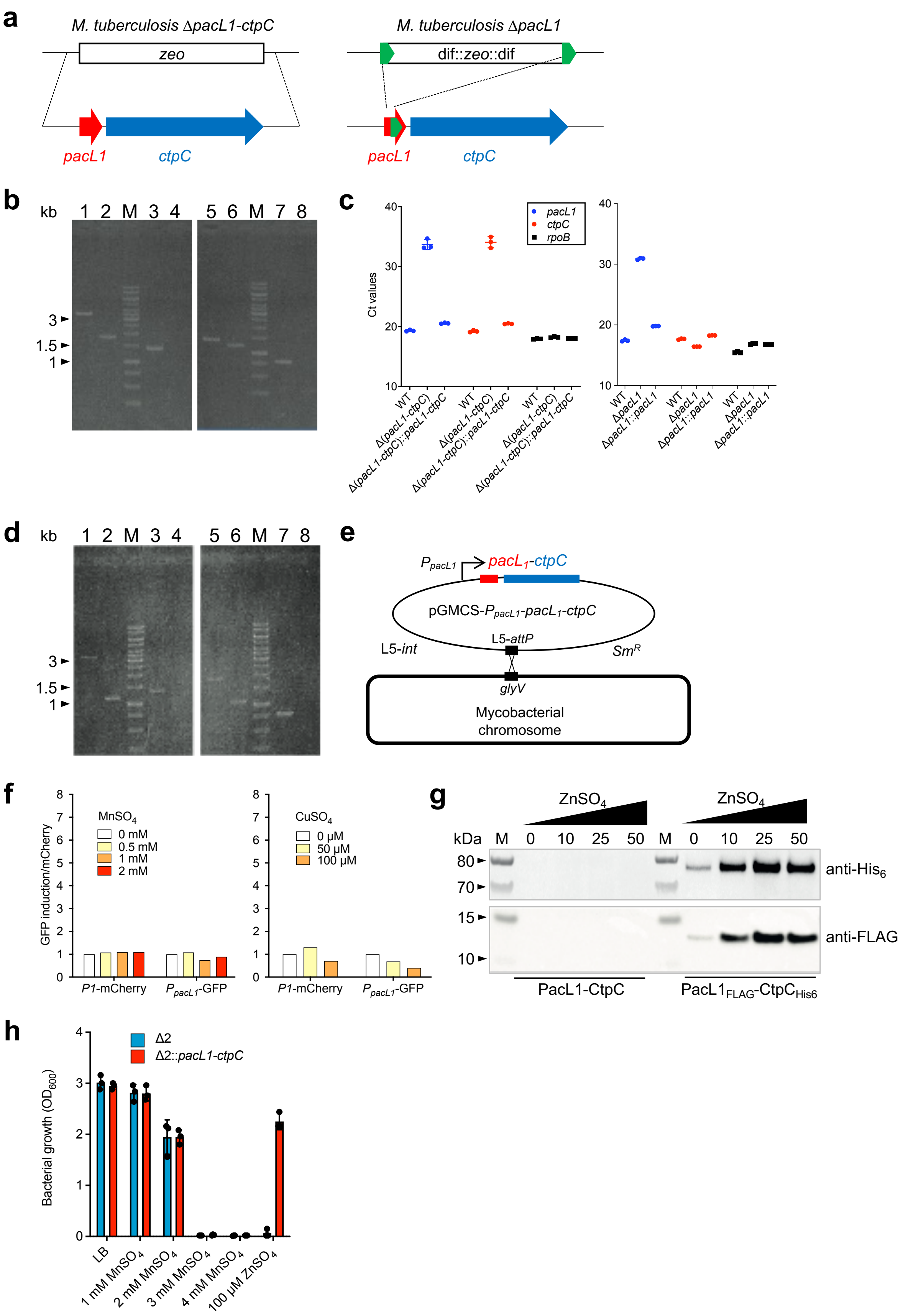

Supplementary Figure 2. Construction of *M. tuberculosis* and *M. smegmatis* mutant and recombinant strains.

(**a**) Schematic representation of the strategy used to inactivate the *pacL1-ctpC* operon and to create an in-phase deletion of *pacL1*.
(**b**) Confirmation of the *pacL1-ctpC* operon inactivation (lanes 1-4) and the in-phase deletion of *pacL1* (lanes 5-8). For *pacL1-ctpC* inactivation, PCR was done with primers producing a 3.5 kb fragment in WT (lane 1), and a 1.9 kb fragment for a complete deletion of the operon (lane 2). The 1.9 kb fragment was verified by Sanger sequencing. Deletion was also confirmed with an internal primer hybridizing to the desired deleted fragment. A fragment of 1.4 kb was produced in WT (lane 3) while no amplification was expected in the mutant strain (lane 4). For *pacL1* deletion, PCR was done with primers producing a 1.4 kb fragment in WT (lane 5), and a 1.2 kb fragment for a complete deletion of *pacL1* (lane 6). The 1.2 kb fragment was verified by Sanger sequencing. Deletion was also confirmed with an internal primer hybridizing to the desired deleted fragment. A fragment of 0.8 kb was produced in WT (lane 7) while no amplification was expected in the mutant strain (lane 8). M, molecular size marker.
(**c**) RT-qPCR quantification (Ct values) of *pacL1*, *ctpC* and *rpoB* expression in *M. tuberculosis* H37Rv (Wild-type, WT), the Δ*pacL1-ctpC* mutant and its complemented strain (left graph), and the Δ*pacL1* mutant and its complemented strain (right graph) in 7H9 medium, which contains 6 mM $ZnSO_4$. Data show mean±s.d of a biological replicate (n=3), and are representative of 2 independent experiments.
(**d**) Confirmation of deletion of the *msmeg_6059-msmeg_6058* operon (lanes 1-4) and of the *msmeg_0755* gene (lanes 5-8). For *msmeg_6059-msmeg_6058* deletion, PCR was done with primers producing a 3.5 kb fragment in WT (lane 1), and a 1.2 kb fragment for a complete deletion of the operon (lane 2). The 1.2 kb fragment was verified by Sanger sequencing. Deletion was also confirmed with an internal primer hybridizing to the desired deleted fragment. A fragment of 1.3 kb was produced in WT (lane 3) while no amplification was expected in the mutant strain (lane 4). For *msmeg_0755* deletion, PCR was done with primers producing a 1.9 kb fragment in WT (lane 5), and a 1.1 kb fragment for a complete deletion of *msmeg_0755* (lane 6). The 1.1 kb fragment was verified by Sanger sequencing. Deletion was also confirmed with an internal primer hybridizing to the desired deleted fragment. A fragment of 0.7 kb was produced in WT (lane 7) while no amplification was expected in the mutant strain (lane 8). M, molecular size marker.
(**e**) Schematic representation of the integrative plasmid used in this study encoding the *pacL1-ctpC* operon under its native promoter P_{pacL1}. L5-*int* and L5-*attP* indicate the gene encoding the integrase and the attachment site of bacteriophage L5, respectively. Sm^R, streptomycin resistance gene.
(**f**) *M. smegmatis* Δ2 was transformed with an integrative vector encoding mCherry under the constitutive promoter *P1*, and GFP under the control of the native promoter of the *pacL1-ctpC* operon (P_{pacL1}). Bacteria were incubated overnight with the indicated concentrations of $MnSO_4$ (left panel) or $CuSO_4$ (right panel), and GFP signal (mean fluorescence intensity, MFI) compared to that of mCherry was measured by flow cytometry. Data (n=1) are representative of 3 independent experiments.
(**g**) Western-blotting analysis of *M. smegmatis* Δ2 expressing recombinant native PacL1 and CtpC (left part), or recombinant FLAG- and His□-tagged PacL1 and CtpC, respectively (right part). Genes are expressed under the control of the P_{pacL1} promoter. Bacteria were grown in 7H9 medium (which contains 6 mM $ZnSO_4$) in the presence of increasing concentrations of additional $ZnSO_4$, as indicated. The upper membrane was treated for His6 immuno-detection and the lower membrane was treated for FLAG immune-detection. M, molecular size marker. Data are representative of 2 independent experiments.
(**h**) *M. smegmatis* strains mc^{2}155 (wild-type, WT), Δ*msmeg_0755* Δ*msmeg_6059-msmeg_6058* (Δ2) or Δ2 transformed with plasmid pGMCS-P_{pacL1}-*paL1-ctpC* were cultivated in LB medium until early stationary phase. At time 0, cultures were inoculated at OD_{600}=0.02 in LB medium

containing the indicated concentrations of $MnSO_4$ or $ZnSO_4$. OD_{600} was measured after 48h of growth at 37°C with shaking. Data show mean±s.d of a biological replicate (n=3), and are representative of 2 independent experiments.
Source data are provided as a Source Data file.

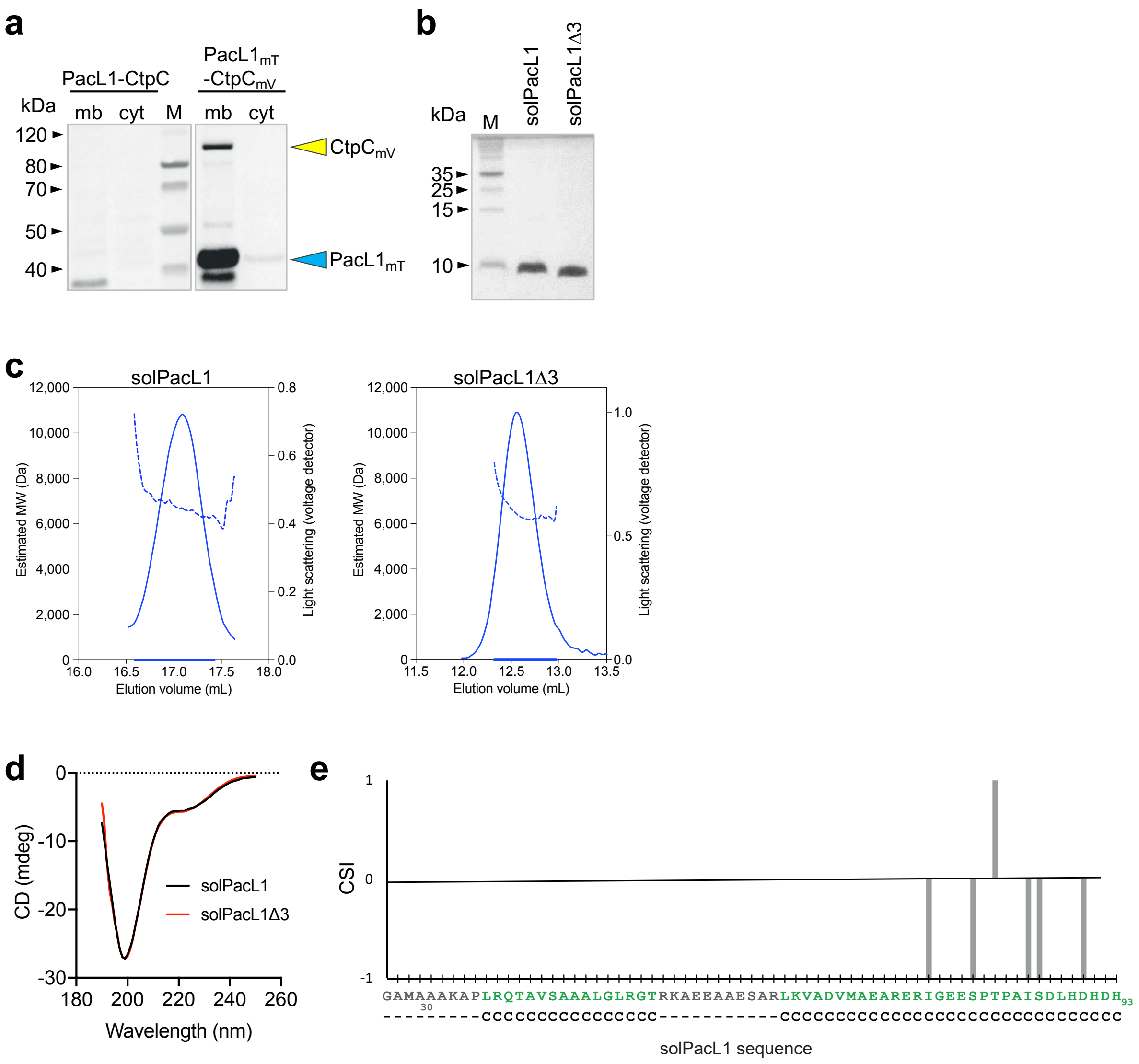

Supplementary Figure 3. PacL1 is a membrane-associated protein and solPacL1 is a disordered protein.

(**a**) Western blotting analysis of soluble and membrane extracts of *M. smegmatis* D2 expressing recombinant native PacL1 and CtpC (left panel), or mTurquoise- and mVenus-tagged PacL1 and CtpC, respectively (right panel). The membrane was treated for mTurquoise and mVenus simultaneous immuno-detection using an anti-eGFP antibody. M, molecular size marker. Data are representative of 2 independent experiments.

(**b**) Coomassie blue stained SDS-polyacrylamide gel (16% acrylamide) of 5 µg of purified solPacL1 and SolPacL1D3. M, molecular size marker. Image is representative of 2 independent experiments.

(**c**) SEC-MALS analysis of 50 µg of purified solPacL1 and SolPacL1D3.

(**d**) Circular dichroism spectra of 30 µM of the purified solPacL1 and SolPacL1D3.

(**e**) Representation of NMR $^{1}H_{\alpha}$ chemical shift index (CSI)[6] according to sequence of solPacl1 that confirms the unfolded-state of solPacl1 under our experimental conditions. Three consecutive non-null CSI values (*i.e.*, +1 or -1) along the sequence is indicative of stable secondary structure elements, respectively β-sheet and α-helix. Annotation; c: random coil and (-): unpredicted (*i.e.*, unassigned residue).

Source data are provided as a Source Data file.

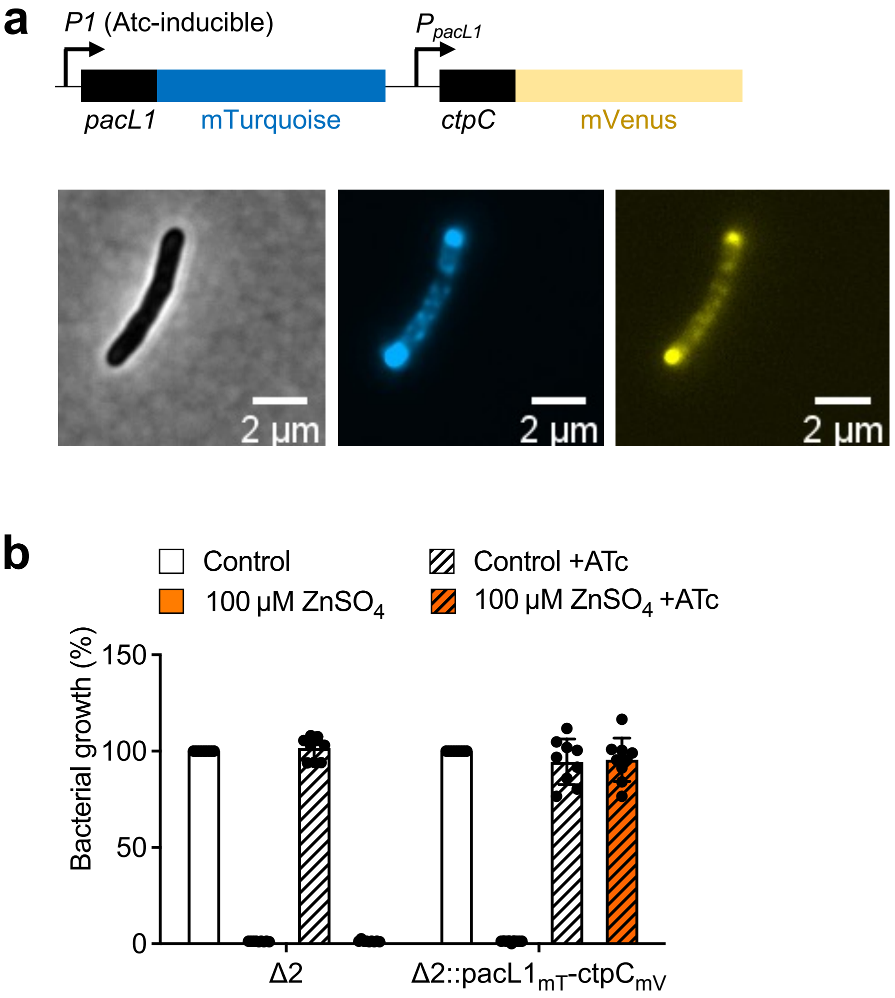

Supplementary Figure 4. Fluorescent fusion PacL1 and CtpC proteins are functional.

(**a**) Epifluorescence microscopy image of *M. smegmatis* Δ2 expressing Atc-inducible mTurquoise-tagged PacL1 and constitutive mVenus-tagged CtpC in the presence of anhydrotetracycline.

(**b**) *M. smegmatis* D2 and the strain used in (A) were cultivated for 24 h in complete 7H9 medium in the absence (Control, white bars) or presence (orange bars) of 100 mM ZnSO4, in the absence (non-hashed bars) or presence (hashed bars) of Atc. Bacterial growth was quantified by turbidity measurement (expressed as % of untreated control). Data show mean±s.d of a biological replicate (n=3), and are representative of 2 independent experiments. Source data are provided as a Source Data file.

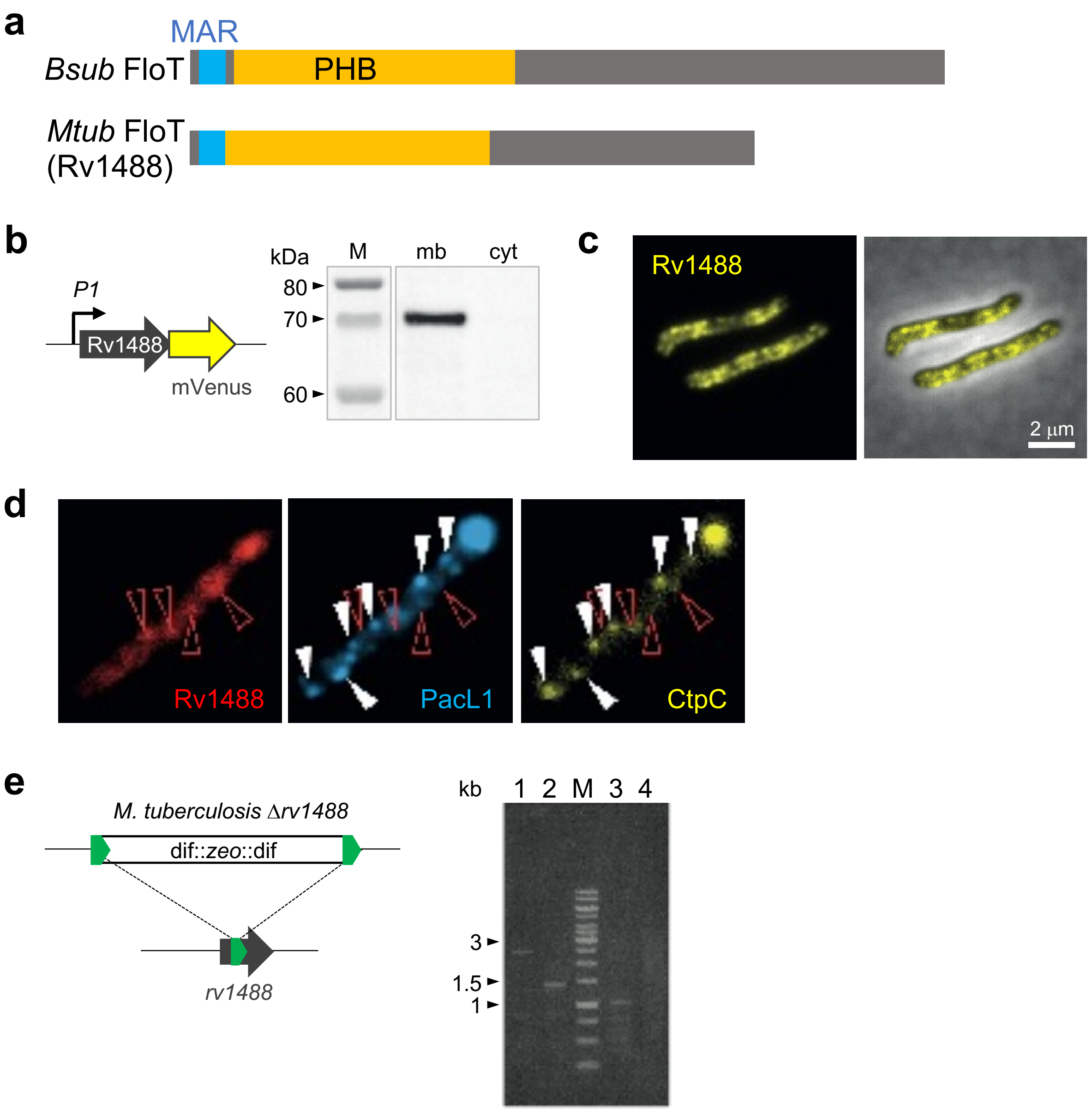

Supplementary Figure 5. PacL1 and CtpC co-localize in membrane microdomains in a flotillin-independent manner.

(**a**) Domain organization of the *B. subtilis* flotillin-like FloT (upper panel), and its *M. tuberculosis* homologue Rv1488 (lower panel). MAR (blue), membrane-associated region. Band_7/prohibitin (PHB) domain (https://www.ebi.ac.uk/interpro/entry/InterPro/IPR036013/) is indicated in yellow.

(**b**) Left panel, *M. smegmatis* was transformed with a vector encoding mVenus-tagged Rv1488 under the control of a tetracycline-inducible promoter *P1* acting constitutively in the absence of TetR protein (left panel). Right panel, Western-blotting analysis of the cytosolic (cyt) and membrane (mb) fractions of the recombinant strain grown in 7H9 medium. The membrane was treated for immuno-detection of mVenus using an anti-GFP antibody. M, molecular size marker. Data are representative of 2 independent experiments.

(**c**) Epifluorescence microscopy examination of *M. smegmatis* expressing mVenus-tagged Rv1488. Images are representative of 3 independent experiments.

(**d**) Epifluorescence microscopy examination of *M. smegmatis* expressing mCherry-tagged Rv1488, mTurquoise-tagged PacL1 and mVenus-tagged CtpC. Red open arrowheads indicate

Rv1488 clusters. White closed arrowheads indicate PacL1/CtpC clusters. Images are representative of 3 independent experiments.

(**e**) Left panel, schematic representation of the Rv1488-KO mutant construction. Right panel, confirmation of deletion of Rv1488. PCR was done with primers producing a 2.4 kb fragment in WT (lane 1), and a 1.4 kb fragment for a complete deletion of the gene (lane 2). The 1.4 kb fragment was verified by Sanger sequencing. Deletion was also confirmed with an internal primer hybridizing to the desired deleted fragment. A fragment of 1.1 kb was produced in WT (lane 3) while no amplification was expected in the mutant strain (lane 4). M, molecular size marker.

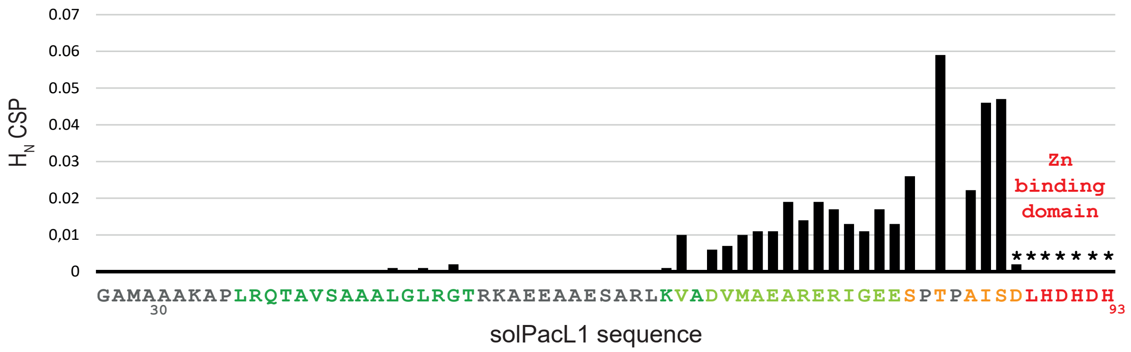

Supplementary Figure 6. solPacL1 binds zinc in its C-terminal end.

Histogram of amide proton chemical shift perturbation (H_N CSP) upon addition of two equivalents of zinc-acetate to a solution of 500 µM in 20 mM MES-d_{13} pH 6.0, 100 mM NaCl at 7 °C. The chemical shift perturbation of amide protons (H_N CSP) induced by zinc binding was measured by the absolute value of the difference in chemical shifts with and without zinc, $|H_{N\ (+)\ Zinc} - H_{N\ (-)\ Zinc}|$. The residues with linewidth broadened close and beyond the detection limit in presence of zinc are annotated by a star (*). The color code of solPacL1 sequence represents the magnitude of the perturbation; red: strong; orange: intermediate; light green and green: unaffected.

Source data are provided as a Source Data file.

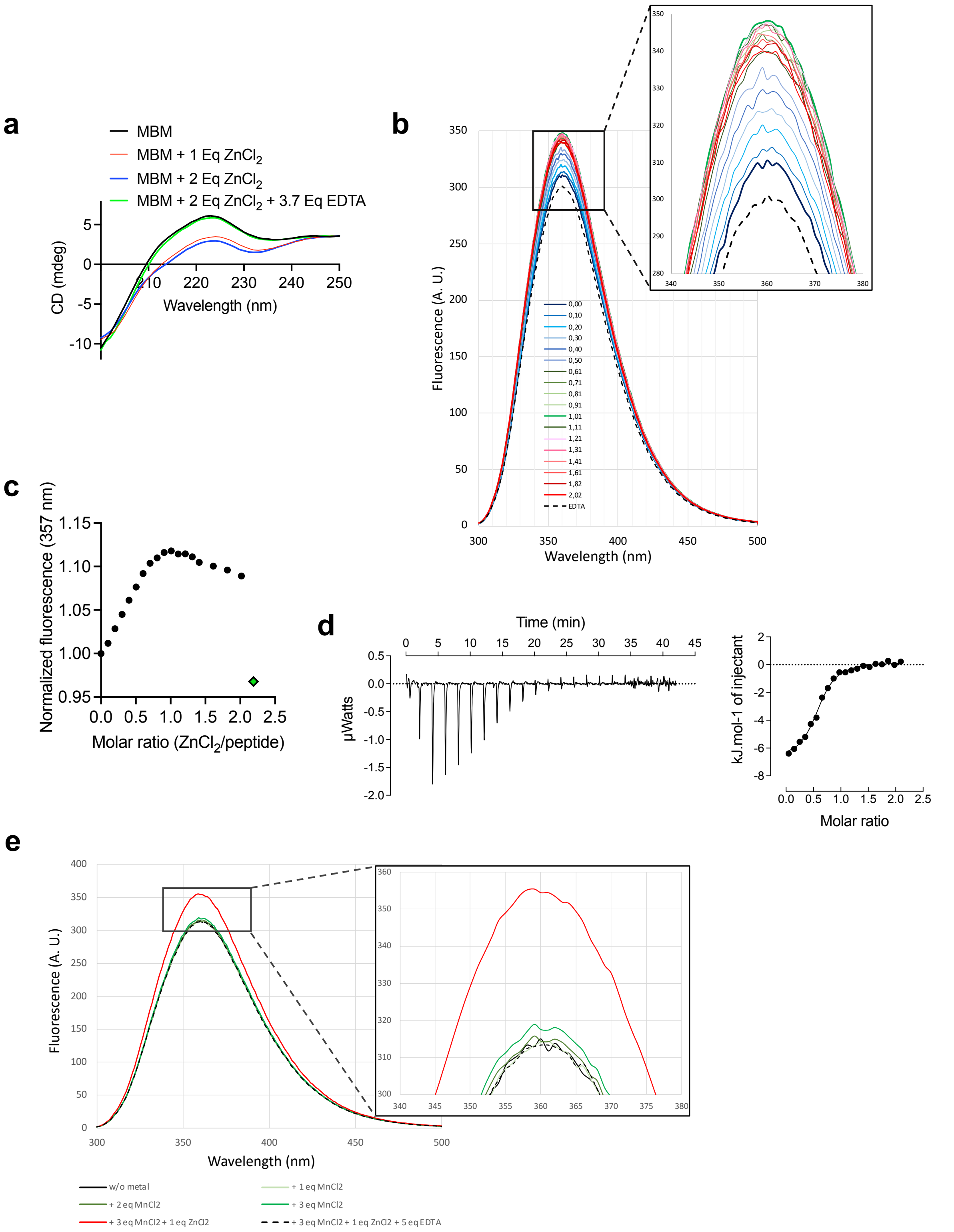

Supplementary Figure 7. The PacL1 C-terminal metal-binding motif (MBM) binds zinc but not manganese.

(**a**) Circular dichroism spectra of Trp-MBM (50 µM) in the presence of 1 and 2 molar equivalents of $ZnCl_2$, with or without 3.7 equivalents of the divalent chelator EDTA.

(**b**) Fluorescence spectra of Trp-MBM as a function of $ZnCl_2$ equivalents (raw data). Black spectrum: without $ZnCl_2$. Color spectra: $ZnCl_2$ equivalents from 0.1 to 2.02 (see color); black

dotted spectrum: with 3 equivalents of EDTA added at the end of the experiment. Inset: zoom on the maximum emission region. The data are representative of three independent experiments.
(**c**) Normalized fluorescence as a function of $ZnCl_2$ to Trp-MBM ratio. Fluorescence changes were normalized to the condition without $ZnCl_2$. Green diamond corresponds to the normalized fluorescence at 3 equivalents of EDTA.
(**d**) Isothermal titration calorimetry data. Thermograms and corresponding titration curves obtained by successive injections of 2 μL of 1.5 mM $Zn[CH_3COO]_2$ into the sample containing 150 μM of Trp-MBM. Molar ratio is defined as the number of Zn^{2+} ions per Trp-MBM. The data are representative of two independent experiments.
(**e**) Fluorescence spectra of Trp-MBM as a function of $MnCl_2$ equivalents (raw data). Black spectrum: without any metals; green spectra: $MnCl_2$ equivalents from 1 to 3 (see color); red spectrum: with 3 equivalents of $MnCl_2$ and 1 equivalent of $ZnCl_2$; black dotted spectrum: with 3 equivalents of $MnCl_2$, 1 equivalent of $ZnCl_2$ and 5 equivalents of EDTA added at the end of the experiment. Inset: zoom on the maximum emission region. The data are representative of one experiment.
Source data are provided as a Source Data file.

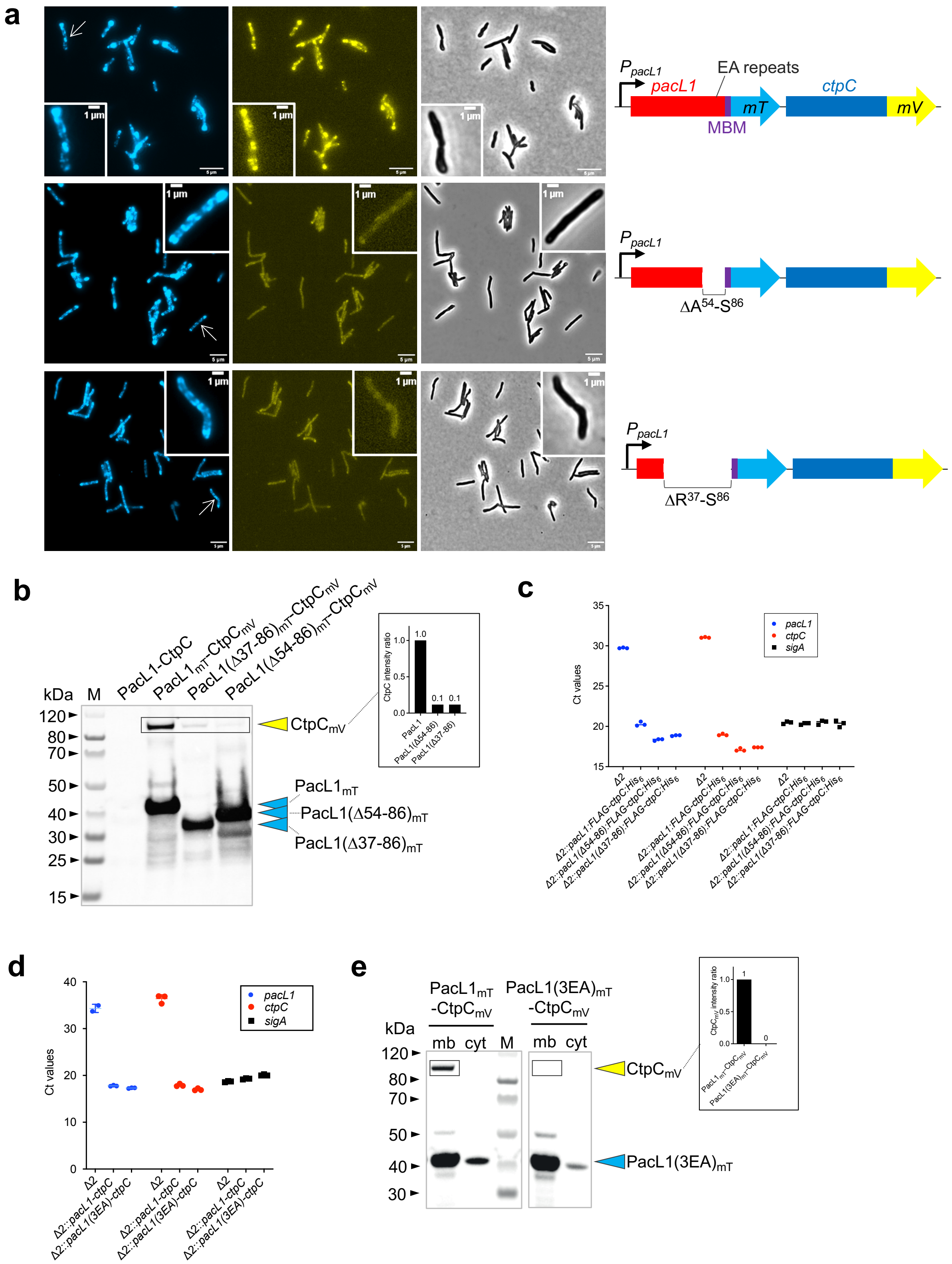

Supplementary Figure 8. PacL1 Glu/Ala repeats are involved in CtpC stabilization in membrane microdomains.

(**a**) Epifluorescence microscopy examination of *M. smegmatis* Δ2 expressing: i/ mTurquoise (mT)-tagged PacL1 and mVenus (mV)-tagged CtpC (upper panels), ii/ mT-PacL1ΔA^{54}-S^{86}, in which the C-terminal MBM is conserved, and mV-CtpC (middle panels), and iii/ mT-

PacL1ΔR^{37}-S^{86}, in which the C-terminal MBM is conserved, and mV-CtpC (lower panels). All genes are expressed under the control of the native P_{PacL1} promoter. All panels of the same color were acquired and displayed with the same parameters. Images are representative of 3 independent experiments.

(**b**) Western-blotting analysis of membrane extracts from strains used in (A) and a control strain expressing non-tagged PacL1 and CtpC. mT and mV were immuno-detected using an anti-eGFP antibody. Blue arrowheads indicate mT-PacL1 or -PacL1 variants; yellow arrowhead indicates CtpC-mV. CtpC-mV protein quantification is displayed in the inset (relative to CtpC-mV amount in the PacL1-mT-expressing strain).

(**c**) RT-qPCR quantification (Ct values) of *pacL1*, *ctpC* and *sigA* expression in strains used in Figure 5A. Data show mean±s.d of a biological replicate (n=3), and are representative of 2 independent experiments.

(**d**) RT-qPCR quantification (Ct values) of *pacL1*, *ctpC* and *sigA* expression in *M. smegmatis* Δ2 (control) and in *M. smegmatis* Δ2 expressing recombinant CtpC and native PacL1 or PacL1 3EA variant, in which E^{55}, E^{59} and E^{71} are mutated into A or V. Data show mean±s.d of a biological replicate (n=3), and are representative of 2 independent experiments.

(**e**) Western-blotting analysis of membrane extracts of cytosolic (cyt) and membrane (mb) fractions from *M. smegmatis* Δ2 expressing recombinant mT-tagged PacL1 and mV-tagged CtpC, or mT-tagged PacL1 3EA variant and mV-tagged CtpC. mT and mV were immuno-detected using an anti-eGFP antibody. Blue arrowhead indicates mT-PacL1 or -PacL1 variant; yellow arrowhead indicates CtpC-mV. CtpC-mV protein quantification is displayed in the inset (relative to CtpC-mV amount in the PacL1-mT-expressing strain). Data are representative of 3 independent experiments.

Source data are provided as a Source Data file.

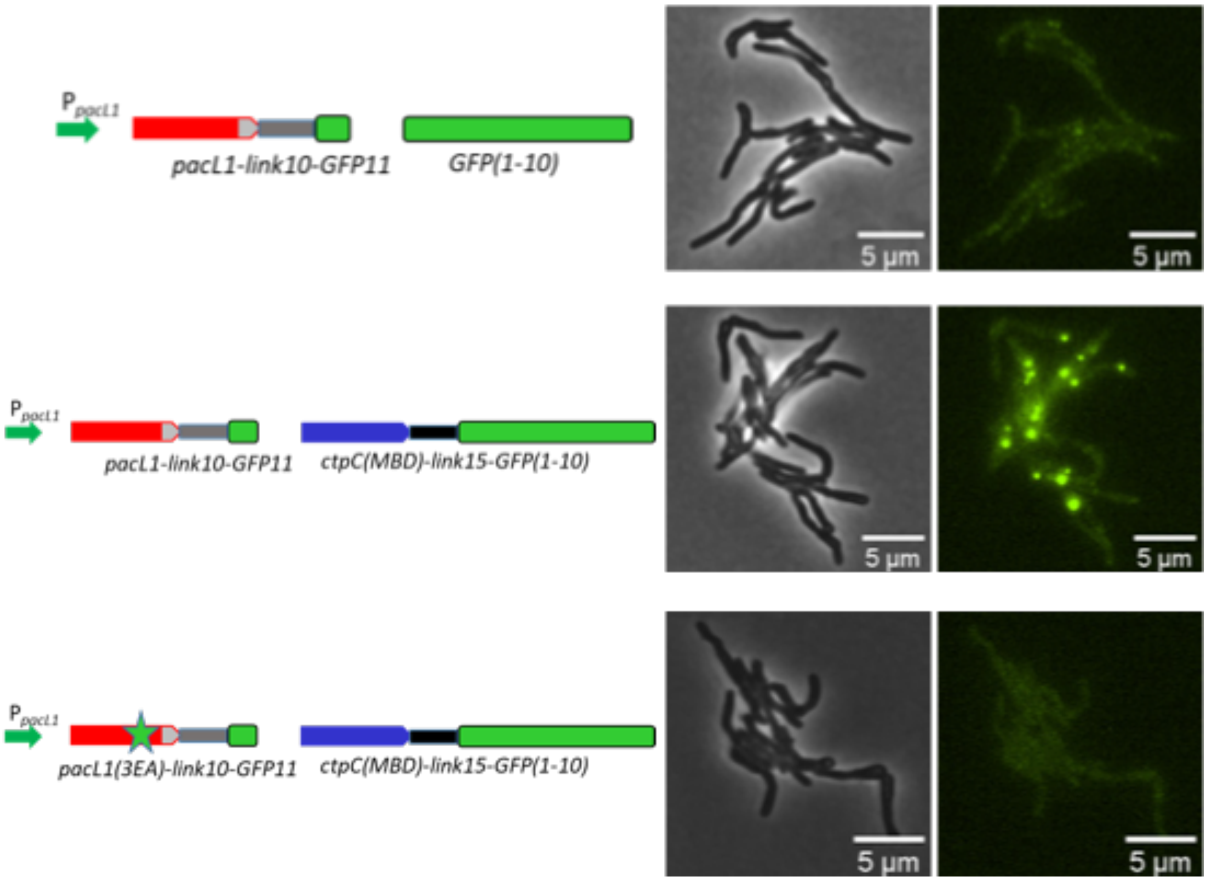

Supplementary Figure 9. Interaction between PacL1 and the CtpC N-terminal region. Bipartite split-GFP experiment using *M. smegmatis* D2 expressing a PacL1-GFP11 fusion protein and control GFP1-10 (top panel), a PacL1-GFP11 fusion protein and the CtpC N-terminal region (M^1-A^{85}) fused to GFP1-10 (middle), or a PacL1(3EA)-GFP11 fusion protein and the CtpC N-terminal region (M^1-A^{85}) fused to GFP1-10 (bottom panel). Bacteria were grown in complete 7H9. Fluorescence was recorded by epifluorescence microscopy. All panels were acquired and displayed with the same parameters. The experiment was performed one time.

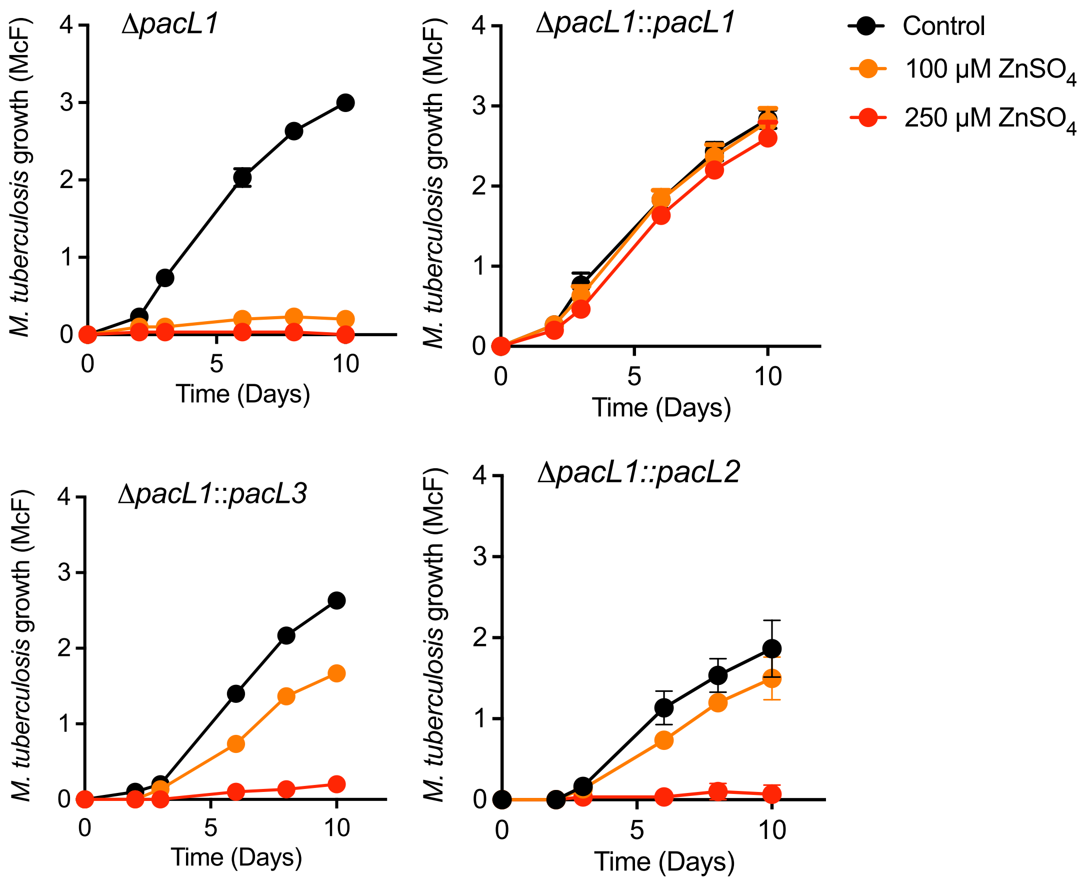

Supplementary Figure 10. Complementation of the Δ*pacL1* mutant with *pacL* genes.

M. tuberculosis Δ*pacL1* complemented with an empty vector (upper left), a PacL1-encoding vector (upper right), a PacL3-encoding vector (lower left) or a PacL2-encoding vector (lower right) were cultivated in complete 7H9 medium containing $ZnSO_4$ at the indicated concentrations (Control, no added zinc). Bacterial growth was quantified by turbidity measurement (expressed in McFarland's units). Data show mean±s.d of a biological replicate (n=3), and are representative of 2 independent experiments.
Source data are provided as a Source Data file.

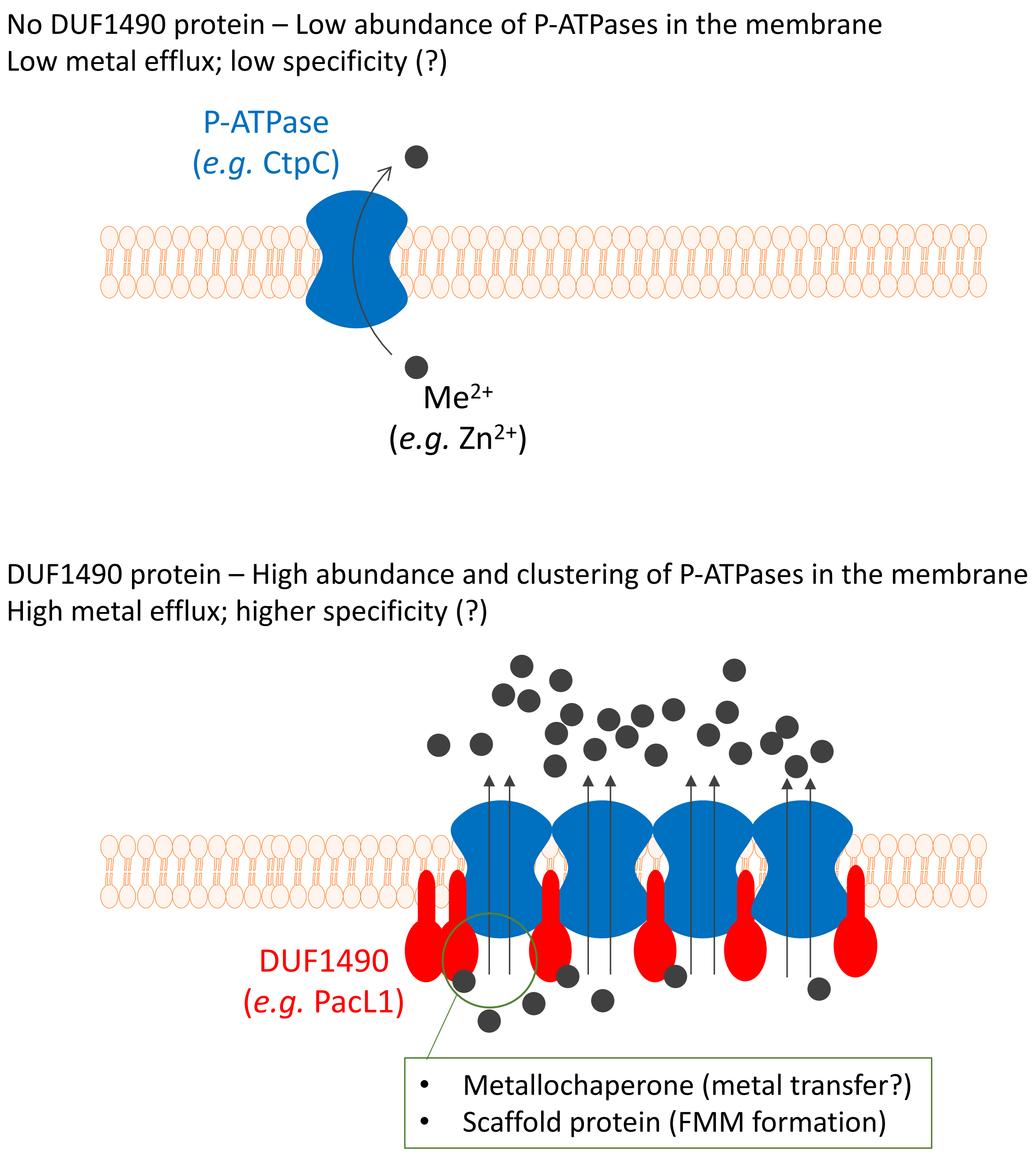

Supplementary Figure 11. Schematic representation of DUF1490 protein function.

In the absence of DUF1490 protein (upper panel), the P-ATPase-encoding gene is expressed but the P-ATPase protein is not stabilized in the plasma membrane. As a consequence, resistance to metal intoxication is poor to null.

In the presence of DUF1490 protein (lower panel), the P-ATPase protein is abundant and stable in the plasma membrane, and assemble into functional microdomains. In addition, DUF1490 protein might increase metal selectivity and P-ATPase activity through its metallochaperone property.

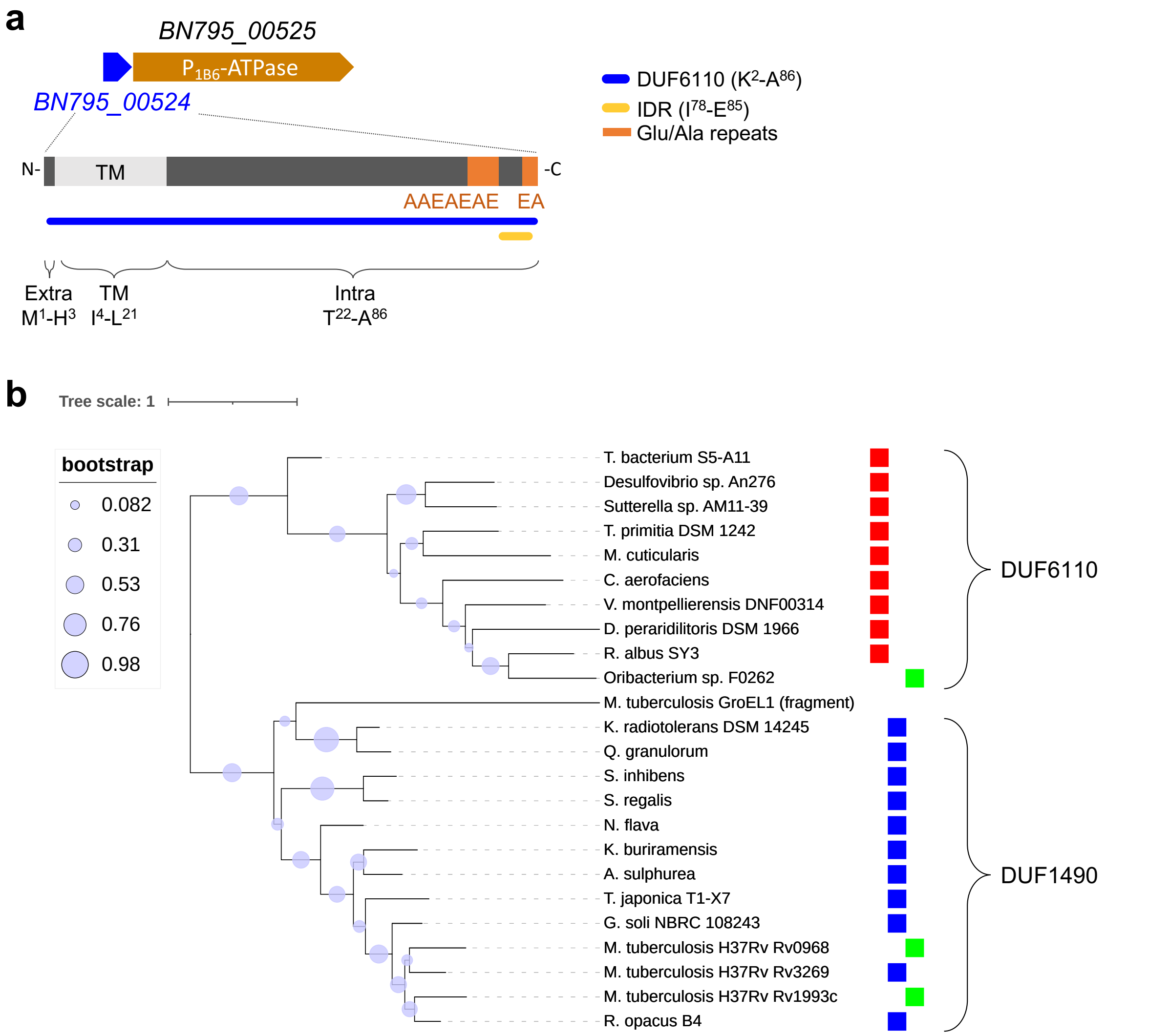

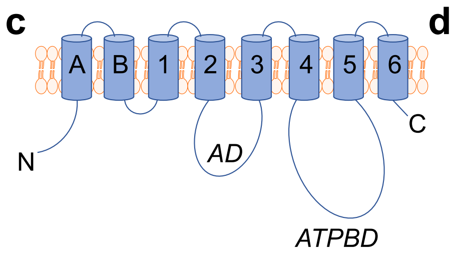

d

	TM4	TM5	TM6	TM5-TM6
P_{1B2}	CPC	Tx_5QNx_7K	$DxGx_7N$	
P_{1B6}	SCA			$Nx_6LGx_2Gx_3Px_4LLHN$
CtpC	CPC	Ax_5QNx_7N	✗	$Nx_5/Gx_2Gx_3Px_4/LHN$

Supplementary Figure 12. DUF6110 proteins are related to DUF1490 proteins.

(**a**) Genetic organization of the *BN795_00524-BN795_00525* module in Firmicutes bacterium CAG:83, and polypeptide composition of DUF6110 protein BN795_00524. Blue tube indicates DUF6110. Yellow tube indicates intrinsically disordered region (IDR) as predicted using D^2P^2 (ref. 7). Protein topology was predicted using TMHMM (TMHMM Server v. 2.0). Extra, extracellular part; TM, transmembrane domain; Intra, intracellular part.

(**b**) Phylogenetic tree of 10 DUF6110 proteins, 13 DUF1490 proteins and the PacL1-matching fragment of *M. tuberculosis* GroEL1 generated by phylogeny.fr[8] (http://www.phylogeny.fr), using MUSCLE, PhyML and TreeDyn, for multiple sequence alignment, tree construction, and tree visualization, respectively. Bootstrap values correspond to approximate Likelihood-Ratio

Test (aLRT). Cognate P_{1B6}-, P_{1B6}-like- and other P-ATPases are indicated by squares in red, blue, and green, respectively.

(**c**) Predicted topology of CtpC according to Constrained Consensus TOPology prediction server (CCTOP)[9]. MBD, putative metal-binding domain with ferredoxin-like fold; AD, actuator domain; ATPBD, ATP-binding domain.

(**d**) Canonical motifs in P_{1B2}- (blue) and P_{1B6}- (red) ATPases[10-12], and their occurrence in CtpC. Highly conserved residues are underlined. Residues in italic are not conserved.

Supplementary uncropped scans:

Fig. 2a

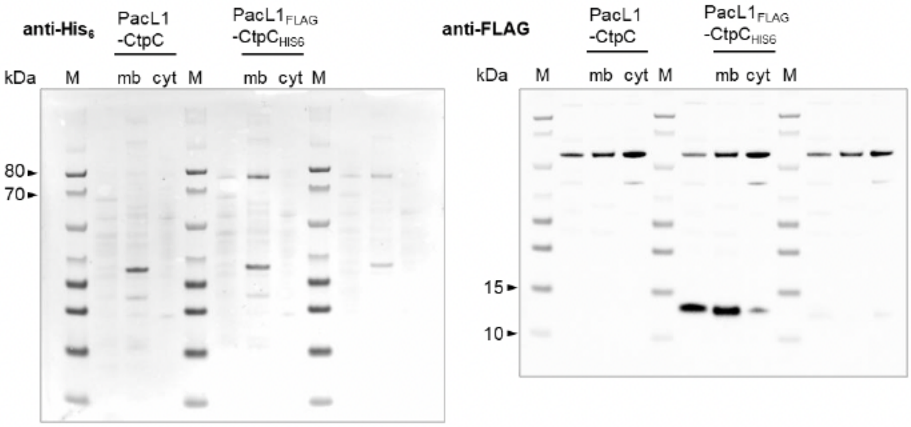

Fig. 2b

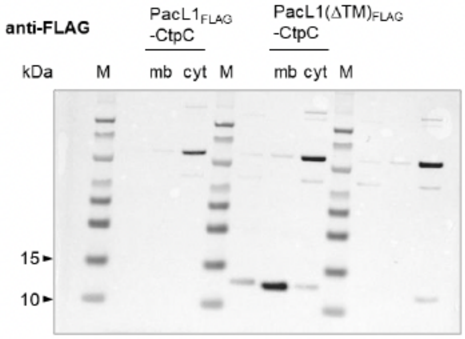

Fig. 5a

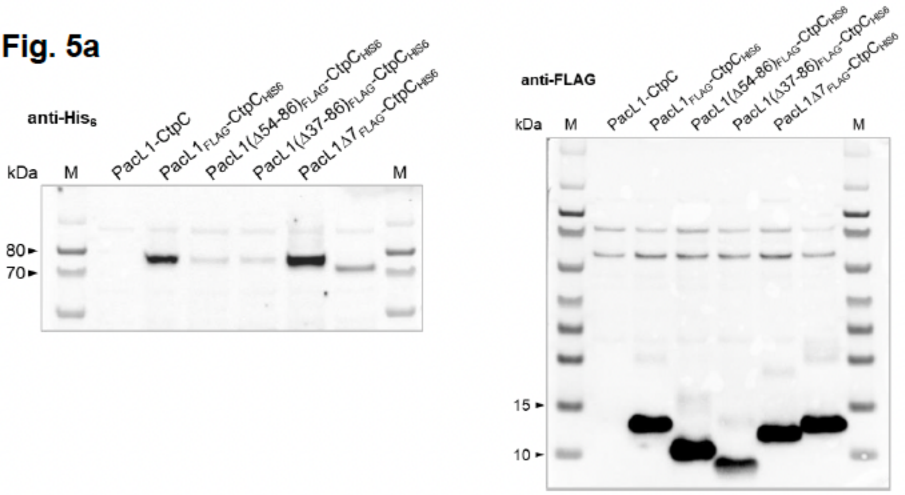

Fig. 5c

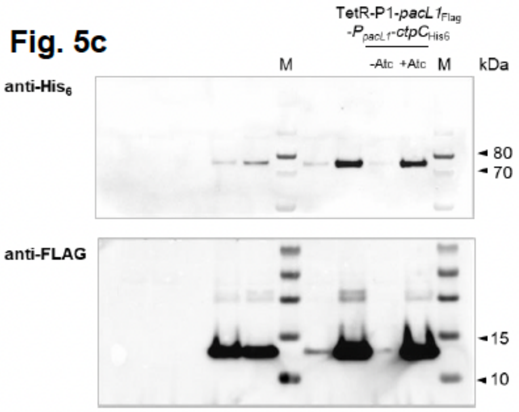

Fig. 6c

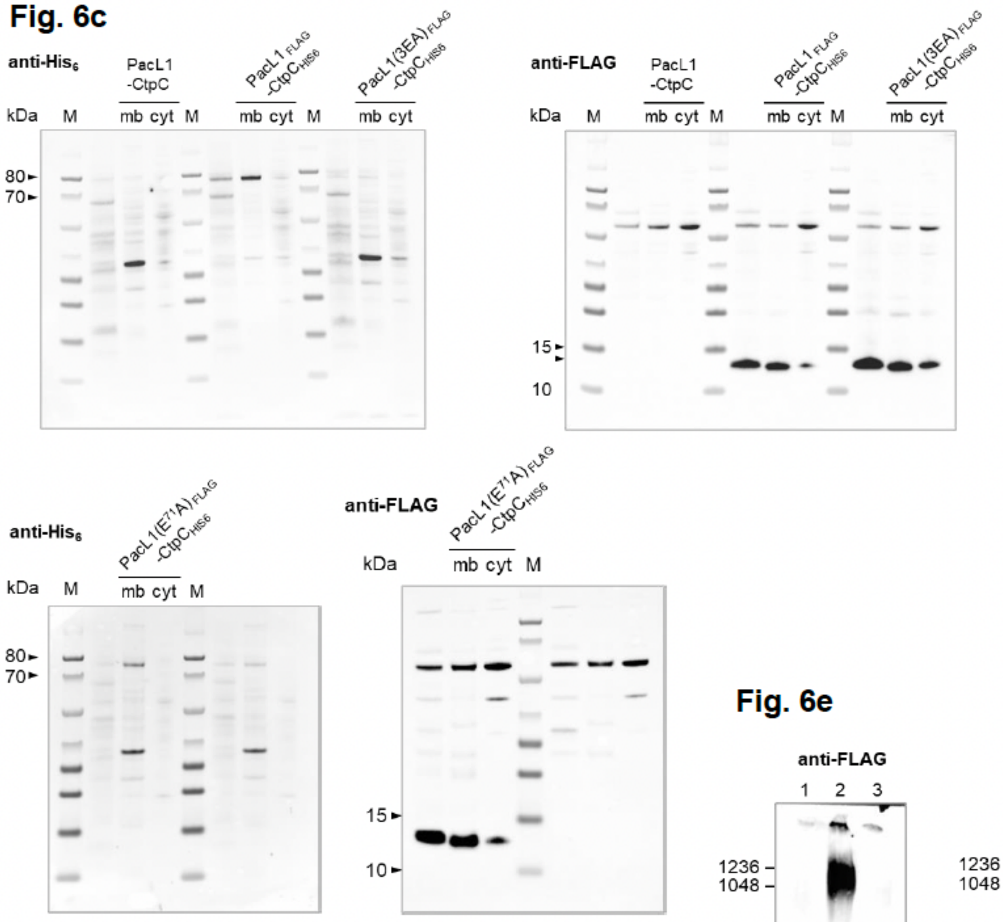

Fig. 6e

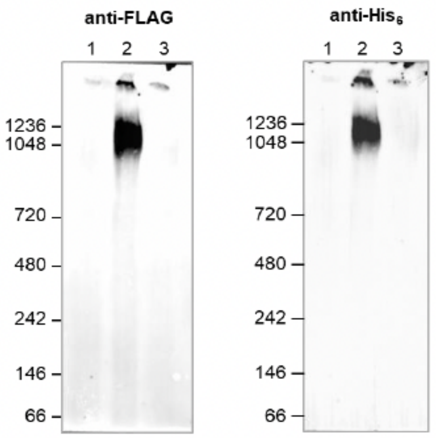

Supplementary Fig. 2g

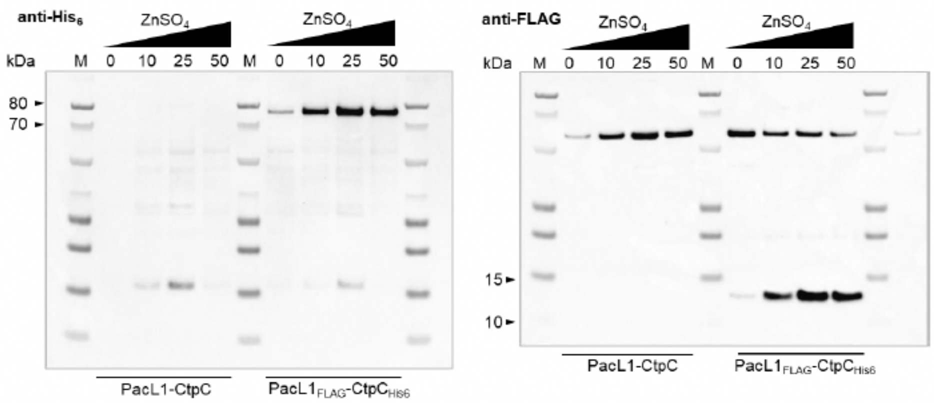

Supplementary Fig. 3a

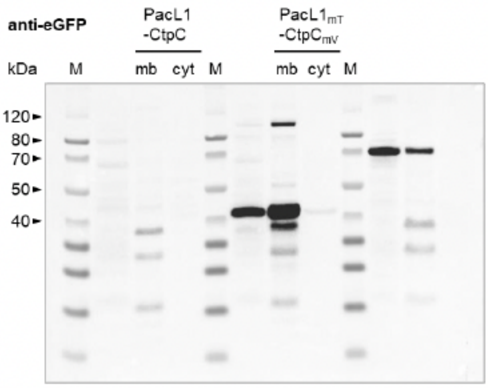

Supplementary Fig. 5b

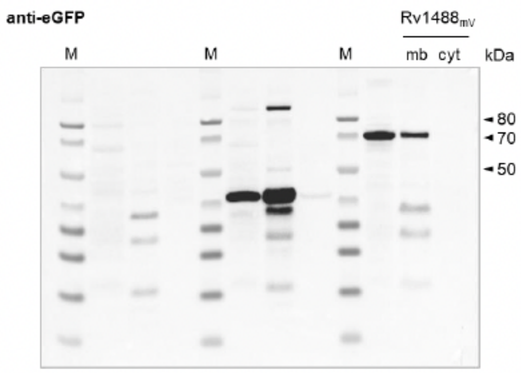

Supplementary Fig. 8b

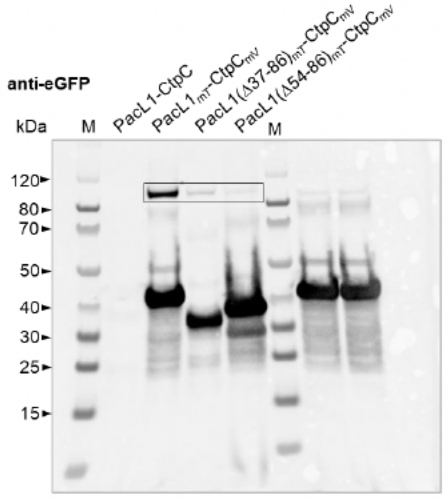

Supplementary Fig. 8e

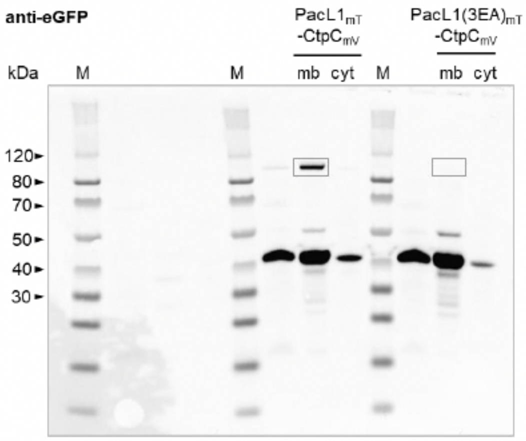

Supplementary Figure 13. Uncropped and unprocessed WB.

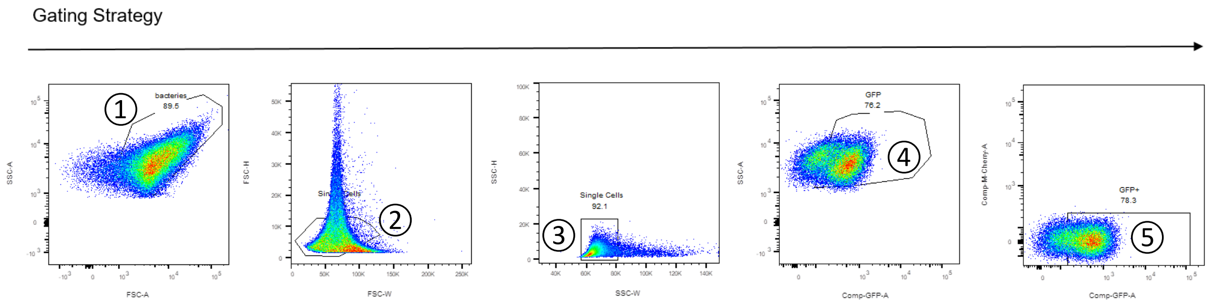

Supplementary Figure 14. Gating strategy.

Bacteria were first sorted based on size (FSC) and structure (SSC) to exclude debries (1), then aggregates were excluded based on FSC and SSC width (W) and height (H) (2, 3). GFP signal was then analyzed *vs*. SSC (4) or *vs*. mCherry, when relevant (5).

Supplementary Table 1. Primers used for the construction of Mycobacterial mutant strains.

***Mycobacterium tuberculosis* strains**	
**Construction of H37Rv Δ(*pacL1-ctpC*)::Zeo^{R}**	
PCR amplification of pacL1 upstream fragment	
3269-Am-Fw	AGCGATACTCGACGATTC
3269-Am-Rv-Zeo	CAGTCGATCCACGTGGAGCATTGCCTGTACCTTTCTTCC
PCR amplification of ctpC downstream fragment	
3270-Zeo-Av-Fw	CCACTGAGCGTCAGACCCACGTGCTCGCCAACAGTTCCCGGTTGATCC
3270-Av-Rev	AGCCTGGCGGTATTGCTCAC
**Construction of H37Rv Δ*pacL1*::*dif4***	
PCR amplification of pacL1 upstream fragment	
3269-Am-Fw	AGCGATACTCGACGATTC
3269-Am-Rv-Zeo2	CAGTCGATCCACGTGGAGGCCATTGCCTGTACCTTTCTTCC
PCR amplification of pacL1 downstream fragment	
3269-Zeo-Av-Fw	CCACTGAGCGTCAGACCCACGTGCTCACCTTGAACTCGCCAGGAC
Seq-ctpC-Rev1	TTCTCGCGCAGGATGAGGCTTG
**Construction of H37Rv ΔRv1488::*dif6***	
PCR amplification of Rv1488 upstream fragment	
Rv1488-Am-Fw	GGCTGACGCGGATCACGCG
Rv1488-Am-Rv-Zeo	CAGTCGATCCACGTGGAGACGGCTCCTTGCACCGGAATTCCTTTC
PCR amplification of Rv1488 downstream fragment	
Rv1488-Zeo-Av-Fw	CCACTGAGCGTCAGACCCACGTGCTCTCAATAGAGTGGTCCGATGAGTGG
Rv1488-Av-Rv	CTAGTGCGGCGTCGACCC
**Construction of H37Rv Δ(*pacL1::dif4 pacL2::dif6 pacL3::dif5*)**	
PCR amplification of pacL2 upstream fragment	
Rv1993c-Am-Fw	CGTGAGTTGGCGGCTCTG
Rv1993c-Am-Rv-Zeo	CAGTCGATCCACGTGGAGGTAACCACGGTCAGTTCTCC
PCR amplification of pacL2 downstream fragment	
Rv1993c-Zeo-Av-Fw	CCACTGAGCGTCAGACCCACGTGCTCCGAGTGACGACTGTAGTTGACGCCGAG
Rv1993c-Av-Rv	CAGCGGATAAGCCCATGCGG
PCR amplification of pacL3 upstream fragment	
Rv0968-Up-Fw	GTTGCTCATCGTCCACGAG
Rv0968-Up-Rv-Zeo	CAGTCGATCCACGTGGAGCCATGCCACACCATCGCGGATG
PCR amplification of pacL3 downstream fragment	
Rv0968-Zeo-Dw-Fw	CCACTGAGCGTCAGACCCACGTGCTCCCACTGACGTTCTTTCTGACACCG
Rv0968-Dw-Rv	GGTAAGCACCGAAGAACATTG
***Mycobacterium smegmatis* strains**	
**Construction of mc^{2}155 Δ(*MSMEG_0755*)::*dif4***	
PCR amplification of MSMEG_0755 upstream fragment	
SMEG0755-Am-Fw	CAGGCGTGCGATTCCGTGCG
SMEG0755-Am-Rv-Zeo	CAGTCGATCCACGTGGAGGTGGCCCGCGCCCATTCC

PCR amplification of MSMEG_0755 downstream fragment	
SMEG0755-Zeo-Av-Fw	CCACTGAGCGTCAGACCCACGTGCTCGCAGGTGCGAGGCCGAGTGAC
SMEG0755-Av-Rv	CGATGGATTCGGCATCCCTTTTGCG
**Construction of mc²155 Δ*(MSMEG_6059-6058)::dif5***	
PCR amplification of MSMEG_6059 upstream fragment	
SMEG6059-Am-Fw	GCAAGCATGAACGTCAATGCG
SMEG6059-Am-Rv-Zeo	CAGTCGATCCACGTGGAGCCATGCCACACCATCGGCCAAGC
PCR amplification of MSMEG_6058 downstream fragment	
SMEG6058-Zeo-Av-Fw	CCACTGAGCGTCAGACCCACGTGCTCCGACTAGGGCGTTCGGCGAACG
SMEG6058-Av-Rv	GTGAGTGGTTTCACTCCG

Supplementary Table 2. **Plasmids used in this study and primers used for their construction.**

Plasmid	Parent vector/ Selection	Cloning technique	Plasmids, Primer pairs or Restriction enzymes used	Reference
pJV53H	pJV53; hygro^R	-	-	[13]
pDE43-MCS	Strepto^R	-	Destination vector for multisite gateway cloning	[14]
pDO41A	Ampi^R	-	Destination vector to construct pEN41 5' entry plasmids for gateway cloning	[14]
pDO12A	Ampi^R	-	Destination vector to construct pEN12 middle entry plasmids for gateway cloning	[14]
pDO23A	Ampi^R	-	Destination vector to construct pEN23 3' entry plasmids for gateway cloning	[14]
pEN41A-T02	Ampi^R	-	Empty 5' entry clone for multisite gateway cloning	[14]
pEN12A-AgeI	Ampi^R	-	Empty middle entry clone for multisite gateway cloning	[14]
pEN23A-MluI	Ampi^R	-	Empty 3' entry clone for multisite gateway cloning	[14]
pEN23A-P_{pacL1}-*pacL1*	pDO23A Ampi^R	Gateway cloning	PCR using H37Rv chromosomal DNA as template and primers clo-B2-3269-Am/clo-B3-3269-Av	This work
pEN23A-P_{pacL1}-*pacL1*-*ctpC*	pDO23A Ampi^R	Gateway cloning	PCR using H37Rv chromosomal DNA as template and primers clo-B2-3269-Am/clo-B3-ctpC-Av	This work
pEN41A-P1-mCherry	pDO41A Ampi^R		Donor of constitutively expressed mCherry reporter for multisite gateway	[15]
pLAM12::GFP	-	-	Source of GFP sequence	[15]
pEN23A-P_{pacL1}-GFP	pDO23A Ampi^R	Gateway cloning	B2-PRv3269-Fw/PRv3269-GFP-Rev to amplify a P_{pacL1} DNA fragment and PRv3269-GFP-Fw/B3-GFP-Rev to amplify a GFP DNA fragment. A fused fragment amplified by two-fragment PCR with B2-PRv3269-Fw/B3-GFP-Rev primers	This work
pGMCS-P1-mCherry	pDE43-MCS Strepto^R	Gateway cloning	pEN41A-P1-mCherry + pEN12A-AgeI + pEN23A-MluI + pDE43-MCS	This work
pGMCS-P1-mCherry-P_{PacL1}-GFP	pDE43-MCS Strepto^R	Gateway cloning	pEN41A-P1-mCherry + pEN12A-AgeI + pEN23A-P_{pacL1}-GFP + pDE43-MCS	This work
pGMCS-P_{pacL1}-*pacL1*	pDE43-MCS Strepto^R	Gateway cloning	pEN41A-T02 + pEN12A-AgeI + pEN23A-P_{pacL1}-*pacL1* + pDE43-MCS	This work

pGMCS-*P*$_{pacL1}$-*pacL1-ctpC*	pDE43-MCS Strepto^R	Gateway cloning	pEN41A-T02 + pEN12A-AgeI + pEN23A-*P*$_{pacL1}$-*pacL1-ctpC* + pDE43-MCS	This work
pGMCS-*P*$_{pacL1}$-*ctpC*	pDE43-MCS Strepto^R	In-fusion cloning	PCR using pGMCS-*P*$_{pacL1}$-*pacL1-ctpC* as template with pGMC 3269 Am Left/pGMC del3269 Right primers and circularization by In-fusion reaction	This work
pGMCS-*P*$_{pacL1}$-*PacL1*Δ3	pDE43-MCS Strepto^R	In-fusion cloning	PCR using pGMCS-*P*$_{pacL1}$-*pacL1* as template with Del3269-Cterm-Left/Del3269-Cterm3-Right primers and circularization by In-fusion reaction	This work
pGMCS-*P*$_{pacL1}$-*PacL1*Δ7	pDE43-MCS Strepto^R	In-fusion cloning	PCR using pGMCS-*P*$_{pacL1}$-*pacL1* as template with Del3269-Cterm-Left/Del3269-Cterm7-Right primers and circularization by In-fusion reaction	This work
pGMCS-*P*$_{pacL1}$-*pacL1*(ΔTM)	pDE43-MCS Strepto^R	In-fusion cloning	PCR using pGMCS-*P*$_{pacL1}$-*pacL1* as template with Del3269-Nterm-Left/Del3269-Nterm7_26-Right primers and circularization by In-fusion reaction	This work
pGMCS-*P*$_{pacL1}$-*pacL1*$_{\mathrm{Flag}}$-*ctpC*	pDE43-MCS Strepto^R	In-fusion cloning	PCR using pGMCS-*P*$_{pacL1}$-*pacL1-ctpC* as template with pGMC-Cter-rv3269 Left #3/3269 Cter Flag-Am-ctpC-Right primers and circularization by In-fusion reaction	This work
pGMCS-*P*$_{pacL1}$-*pacL1*$_{\mathrm{Flag}}$-*ctpC*$_{\mathrm{His6}}$	pDE43-MCS Strepto^R	In-fusion cloning	PCR using pGMCS-*P*$_{pacL1}$-*pacL1*$_{\mathrm{Flag}}$-*ctpC* as template with pGMC-Cter-rv3269 Left #1/ctpC Cter His-Av-attB3-Right primers and circularization by In-fusion reaction	This work
pGMCS-*P*$_{pacL1}$-*pacL1*(Δ7)$_{\mathrm{Flag}}$-*ctpC*$_{\mathrm{His6}}$	pDE43-MCS Strepto^R	In-fusion cloning	PCR using pGMCS-*P*$_{pacL1}$-*pacL1*$_{\mathrm{Flag}}$-*ctp C*$_{\mathrm{His6}}$ as template with pGMC-Cter-ctpC-left #1/ctpC Cter His-Av-attB3-Right primers and circularization by In-fusion reaction	This work
pGMCS-*P*$_{pacL1}$-*pacL1*(ΔTM)$_{\mathrm{Flag}}$-*ctpC*	pDE43-MCS Strepto^R	In-fusion cloning	PCR using pGMCS-*P*$_{pacL1}$-*pacL1*$_{\mathrm{Flag}}$-*ctp C* as template with Del3269-Nterm-Left/Del3269-Nterm7_26-Right primers and circularization by In-fusion reaction	This work
pJYB234	Cam^R	-	Source of mVenus sequence	[16]
pJYB240	Cam^R	-	Source of mTurquoise sequence	[17]
pGMCS-*P*$_{pacL1}$-*pacL1*$_{\mathrm{mT}}$-*ctpC*	pDE43-MCS Strepto^R	In-fusion cloning	m-Turquoise coding fragment amplified with pJYB240 template and mTurquoise-Fw #3/mTurquoise-Rv#3 primers cloned by In-fusion reaction in the backbone amplified from pGMCS-	This work

			P_{pacL1}-*pacL1-ctpC* with pGMC-Cter-rv3269 Left #3/pGMC-Am-ctpC Right #3	
pGMCS-P_{pacL1}(ΔPB)-*pacL1*$_{\text{mT}}$-*ctpC*	pDE43-MCS Strepto^{R}	In-fusion cloning	PCR using pGMCS-P_{pacL1}-*pacL1*$_{\text{mT}}$-*ctpC* with Inf-ctpC-MutPB-L1/Inf-ctpC-MutPB-R1 primers and circularization by by In-fusion reaction	This work
pGMCS-P_{pacL1}-*pacL1-ctpC*$_{\text{mV}}$	pDE43-MCS Strepto^{R}	In-fusion cloning	m-Venus coding fragment amplified with pJYB234 template and mVenus-Fw #1/mVenus-Rv#1 primers cloned by In-fusion reaction in the backbone amplified from pGMCS-P_{pacL1}-*pacL1-ctpC* with pGMC-Cter-ctpC Left #1/pGMC-Av-attB3-Right #1	This work
pGMCS-P_{pacL1}-*pacL1*$_{\text{mT}}$-*ctpC*$_{\text{mV}}$	pDE43-MCS Strepto^{R}	In-fusion cloning	m-Turquoise coding fragment amplified with pJYB240 template and mTurquoise-Fw #3/mTurquoise-Rv#3 primers cloned by In-fusion reaction in the backbone amplified from pGMCS-P_{pacL1}-*pacL1-ctpC*$_{\text{mV}}$ with pGMC-Cter-rv3269 Left #3/pGMC-Am-ctpC Right #3	This work
pGMCS-P_{pacL1}-pacL1(ΔTM)$_{\text{mT}}$-*ctpC*	pDE43-MCS Strepto^{R}	In-fusion cloning	PCR using pGMCS-P_{pacL1}-*pacL1*$_{\text{mT}}$-*ctpC* as template with Del3269-Nterm-Left/Del3269-Nterm7_26-Right primers and circularization by In-fusion reaction	This work
pGMCS-P_{pacL1}-*pacL1*$_{\text{mT}}$-Rv1488$_{\text{mV}}$	pDE43-MCS Strepto^{R}	In-fusion cloning	Rv1488 coding fragment amplified with H37Rv genomic DNA and infus Rv1488 Fw/infus mVenus Rv1488 Rv primers cloned by In-fusion reaction in the backbone amplified from pGMCS-P_{pacL1}-*pacL1*$_{\text{mT}}$-*ctpC*$_{\text{mV}}$ with primers mT av left/infus backbone mVenus Right	This work
pEN41A-P1-mCherry	pDO41A Ampi^{R}	-	-	[15]
pEN41A-P1-Rv1488$_{\text{mC}}$	pDO41A Ampi^{R}	In-fusion cloning	Rv1488 coding fragment amplified from pGMCS-P_{pacL1}-*pacL1*$_{\text{mT}}$-Rv1488$_{\text{mV}}$ and infus P1 Rv1488 Fw/pGMC-3269-FLUO-ctpC Left primers cloned by In-fusion reaction into the backbone amplified from pEN41A-P1-mCherry with mCherry Fw2/Infus pEN41A-P1_x_mCherry Right primers	This work
pGMCS-P1-Rv1488$_{\text{mC}}$-P_{pacL1}-	pDE43-MCS Strepto^{R}	In-fusion cloning	Rv1488$_{\text{mC}}$ fragment amplified with pEN41A-P1-Rv1488$_{\text{mC}}$ and infus P1 Rv1488 Fw (pGMC)/infus mCherry	This work

pacL1$_{\mathrm{mT}}$-*ctpC*$_{\mathrm{mV}}$			Rv cloned by In-fusion reaction in the backbone amplified from pGMCS-P_{pacL1}-*pacL1*$_{\mathrm{mT}}$-*ctpC*$_{\mathrm{mV}}$ with pGMC-12-left/pGMC-12-right	
pGMCS-P_{pacL1}-*pacL1*(Δ37-86)$_{\mathrm{Flag}}$-*ctpC*$_{\mathrm{His6}}$	pDE43-MCS Strepto^{R}	In-fusion cloning	PCR using pGMCS-P_{pacL1}-*pacL1*$_{\mathrm{Flag}}$-*ctpC*$_{\mathrm{His6}}$ as template with Inf-3269Del(37-86)-left/Inf-3269C7-right primers and circularization by In-fusion reaction	This work
pGMCS-P_{pacL1}-*pacL1*(Δ54-86)$_{\mathrm{Flag}}$-*ctpC*$_{\mathrm{His6}}$	pDE43-MCS Strepto^{R}	In-fusion cloning	PCR using pGMCS-P_{pacL1}-*pacL1*$_{\mathrm{Flag}}$-*ctpC*$_{\mathrm{His6}}$ as template with Inf-3269Del(53-86)-left/Inf-3269C7-right primers and circularization by In-fusion reaction	This work
pGMCS-P_{pacL1}-*pacL1*(Δ37-86)$_{\mathrm{mT}}$-*ctpC*$_{\mathrm{mV}}$	pDE43-MCS Strepto^{R}	In-fusion cloning	PCR using pGMCS-P_{pacL1}-*pacL1*$_{\mathrm{mT}}$-*ctpC*$_{\mathrm{mV}}$ as template with Inf-3269Del(37-86)-left/Inf-3269C7-right primers and circularization by In-fusion reaction	This work
pGMCS-P_{pacL1}-*pacL1*(Δ54-86)$_{\mathrm{mT}}$-*ctpC*$_{\mathrm{mV}}$	pDE43-MCS Strepto^{R}	In-fusion cloning	PCR using pGMCS-P_{pacL1}-*pacL1*$_{\mathrm{mT}}$-*ctpC*$_{\mathrm{mV}}$ as template with Inf-3269Del(53-86)-left/Inf-3269C7-right primers and circularization by In-fusion reaction	This work
pGMCS-P_{pacL1}-*pacL1*(E^{55}V)-*ctpC*	pDE43-MCS Strepto^{R}	In-fusion cloning	PCR using pGMCS-P_{pacL1}-*pacL1-ctpC* as template with subs EA 55-59 Left/subs EA 55 Right primers and circularization by In-fusion reaction	This work
pGMCS-P_{pacL1}-*pacL1*(E^{59}A)-*ctpC*	pDE43-MCS Strepto^{R}	In-fusion cloning	PCR using pGMCS-P_{pacL1}-*pacL1-ctpC* as template with subs EA 59 Left/subs EA 59 Right primers and circularization by In-fusion reaction	This work
pGMCS-P_{pacL1}-*pacL1*(E^{71}A)-*ctpC*	pDE43-MCS Strepto^{R}	In-fusion cloning	PCR using pGMCS-P_{pacL1}-*pacL1-ctpC* as template with Rv3269(E71A) subst Left/Rv3269(E71A) subst Right primers and circularization by In-fusion reaction	This work
pGMCS-P_{pacL1}-*pacL1*(3EA)-*ctpC*	pDE43-MCS Strepto^{R}	In-fusion cloning	PCR using pGMCS-P_{pacL1}-*pacL1(E*^{71}*A)-ctpC* as template with subs EA 55-59 Left/subs EA 55-59 Right primers and circularization by In-fusion reaction	This work
pGMCS-P_{pacL1}-*pacL1*(E^{71}A)$_{\mathrm{Flag}}$-*ctpC*$_{\mathrm{His6}}$	pDE43-MCS Strepto^{R}	In-fusion cloning	PCR using pGMCS-P_{pacL1}-*pacL1*$_{\mathrm{Flag}}$-*ctpC*$_{\mathrm{His6}}$ as template with Rv3269(E71A) subst Left/Rv3269(E71A) subst Right primers and circularization by In-fusion reaction	This work

pGMCS-P_{pacL1}-*pacL1*(3EA)$_{\text{Flag}}$-*ctpC*$_{\text{His6}}$	pDE43-MCS Strepto^{R}	In-fusion cloning	PCR using pGMCS-P_{pacL1}-*pacL1*(E71A) $_{\text{Flag}}$-*ctpC*$_{\text{His6}}$ as template with subs EA 55-59 Left/subs EA 55-59 Right primers and circularization by In-fusion reaction	This work
pGMCS-P_{pacL1}-*pacL1*(3EA)$_{\text{mT}}$-*ctpC*$_{\text{mV}}$	pDE43-MCS Strepto^{R}	In-fusion cloning	PCR using pGMCS-P_{pacL1}-*pacL1*(E71A) $_{\text{mT}}$-*ctpC*$_{\text{mV}}$ as template with subs EA 55-59 Left/subs EA 55-59 Right primers and circularization by In-fusion reaction	This work
pGMCS-P_{pacL1}-*pacL2*	pDE43-MCS Strepto^{R}	In-fusion cloning	*pacL2* fragment amplified with H37Rv chromosomal DNA and Inf-1993c-Am/Inf-1993c-Av cloned by In-fusion reaction in the backbone amplified from pGMCS-P_{pacL1}-*pacL1* with Inf-Rv3269-left/Inf-Rv3269-right	This work
pGMCS-P_{pacL1}-*pacL3*	pDE43-MCS Strepto^{R}	In-fusion cloning	*pacL3* fragment amplified with H37Rv chromosomal DNA and Inf-0968-Am/Inf-0968-Av cloned by In-fusion reaction in the backbone amplified from pGMCS-P_{pacL1}-*pacL1* with Inf-Rv3269-left/Inf-Rv3269-right	This work
pGMCS-P_{pacL1}-*pacL2-ctpC*	pDE43-MCS Strepto^{R}	In-fusion cloning	*pacL2* fragment amplified with H37Rv chromosomal DNA and Inf-1993-Am/Inf-1993-Av cloned by In-fusion reaction in the backbone amplified from pGMCS-P_{pacL1}-*pacL1-ctpC* with Inf-Rv3269-left/Inf-Rv3269-right	This work
pGMCS-P_{pacL1}-*pacL2*(MBM)-*ctpC*	pDE43-MCS Strepto^{R}	In-fusion cloning	*pacL2*(MBM) fragment amplified with H37Rv chromosomal DNA and Inf-1993-Am/Inf-1993MBM-Av cloned by In-fusion reaction in the backbone amplified from pGMCS-P_{pacL1}-*pacL1-ctpC* with Inf-Rv3269-left/Inf-Rv3269-right	This work
pGMCS-P_{pacL1}-*pacL3-ctpC*	pDE43-MCS Strepto^{R}	In-fusion cloning	*pacL3* fragment amplified with H37Rv chromosomal DNA and Inf-0968-Am/Inf-0968-Av cloned by In-fusion reaction in the backbone amplified from pGMCS-P_{pacL1}-*pacL1-ctpC* with Inf-Rv3269-left/Inf-Rv3269-right	This work
pGMCS-P_{pacL1}-*pacL1*$_{\text{mT}}$-*pacL2*$_{\text{mV}}$	pDE43-MCS Strepto^{R}	In-fusion cloning	*pacL2* fragment amplified with H37Rv chromosomal DNA and Rv1993c-XFP-Fw/ Rv1993c-XFP-Rv cloned by In-fusion reaction in the backbone amplified from pGMCS-P_{pacL1}-*pacL1*$_{\text{mT}}$-*ctpC*$_{\text{mV}}$ with mT av left/infus backbone mVenus Right primers	This work

pGMCS-*P*$_{pacL1}$-*pacL1*$_{mT}$-*pacL3*$_{mV}$	pDE43-MCS Strepto^{R}	In-fusion cloning	*pacL3* fragment amplified with H37Rv chromosomal DNA and Rv0968-XFP-Fw/ Rv0968-XFP-Rv cloned by In-fusion reaction in the backbone amplified from pGMCS-*P*$_{pacL1}$-*pacL1*$_{mT}$-*ctpC*$_{mV}$ with mT av left/infus backbone mVenus Right	This work
pGMCS-TetR-P1-MbcAT	pDE43-MCS StreptoR	-	-	[15]
pGMCS-TetR-P1-*pacL1*$_{Flag}$-*P*$_{pacL1}$-*ctpC*$_{His6}$	pDE43-MCS StreptoR	In-fusion cloning	A TetR-encoding DNA fragment was amplified using pGMCS-TetR-P1-MbcAT as template and TetR-Am-Fus-P1/TetR-Av-Rv primers. A P1 promoter fragment was amplified using pGMCS-TetR-P1-MbcAT as template and Inf-P1-left/Tet-P1-Fus-Fw primers. A PacL1$_{Flag}$-encoding fragment was amplified using pGMCS-*P*$_{pacL1}$-*pacL1*$_{Flag}$-*ctpC*$_{His6}$ as template with Rv3269-P1-Fus-Fw/Rv3269-Flag-Rev primers. These fragments were cloned by In-Fusion reaction in the backbone amplified from pGMCS-*PpacL1-pacL1*$_{Flag}$-*ctpC*$_{His}$ with ctpC-B2-left/ctpC-B2-right primers.	This work
pGMCS-TetR-P1-*pacL1*$_{mT}$-*P*$_{pacL1}$-*ctpC*$_{mV}$	pDE43-MCS StreptoR	In-fusion cloning	A TetR-encoding DNA fragment was amplified using pGMCS-TetR-P1-MbcAT as template and TetR-Am-Fus-P1/TetR-Av-Rv primers. A P1 promoter fragment was amplified using pGMCS-TetR-P1-MbcAT as template and Inf-P1-left/Tet-P1-Fus-Fw primers. A PacL1$_{mT}$-encoding fragment was amplified using pGMCS-*P*$_{pacL1}$-*pacL1*$_{mT}$-*ctpC*$_{mV}$ as template with Rv3269-P1-Fus-Fw/Rv3269-mT-Rev primers. These fragments were cloned by In-Fusion reaction in the backbone amplified from pGMCS-*P*$_{pacL1}$-*pacL1*$_{mT}$-*ctpC*$_{mV}$ with ctpC-B2-left/ctpC-B2-right primers.	This work
pProEx-Htb	Ampi^{R}		Expression vector with IPTG-inducible trc promoter, His6 tag and TEV cleavage site.	Invitrogen
pProEx-Htb-SolPacL1	pProEx-Htb; Ampi^{R}	Restriction site cloning	A DNA fragment encoding SolPacL1 was amplified using H375v chromosomal DNA and	This work

			SolPacL1Fw/SolPacL1Rv primers, cut with *NcoI* and *HindIII* and ligated in pProEx-Htb cut with *NcoI* and *HindIII*	
pProEx-Htb-SolPacL1Δ3	pProEx-Htb; Ampi^{R}	Restriction site cloning	pProEx-Htb-SolPacL1 DNA was amplified with fragment encoding SolPacL1 was amplified with SolPacL1Δ3Fw/SolPacL1Δ3Rv primers, cut with *HindIII* and self-ligated	This work
pET GFP10-FRB-IRBS-FKBPGFP11	Spectino^{R}	-	-	[18]
pET p15 ori GFP1-10 OPT	Kana^{R}	-	-	[18]
pGMCS-P_{pacL1}-*pacL1*,GFP11-Ø-GFP(1-10)	pDE43-MCS Strepto^{R}	In-fusion cloning	A GFP11 DNA fragment was amplified using pET GFP10-FRB-IRBS-FKBPGFP11 as template and 3269 gfp11 L2 Fw /3269 gfp11 Rv primers. A GFP1-10 DNA fragment was amplified using pET p15 ori GFP1-10 OPT as template and GFP1-10 Fw inf ctpC / GFP1-10 Rv L2 inf ctpc primers. These fragments were cloned by In-Fusion reaction in the backbone amplified from pGMCS-P_{pacL1}-*pacL1-ctpC* with ctpC am left2 / ctpC am right primers.	This work
pGMCS-P_{pacL1}-*pacL1*,GFP11-*ctpC*(MBD)-GFP(1-10)	pDE43-MCS Strepto^{R}	In-fusion cloning	A MBD DNA fragment was amplified using pGMCS-P_{pacL1}-*pacL1-ctpC* as template and inf CtpC-Nter #1 / inf-CtpC-MBD-L15 primers. This fragment was cloned by In-Fusion reaction in the backbone amplified from pGMCS-pacL1,GFP11-Ø-GFP(1-10) with CtpC am left2 / FL15 left primers.	This work
pGMCS-P_{pacL1}-*pacL1*($E^{71}A$),GFP11-*ctpC*(MBD)-GFP(1-10)	pDE43-MCS Strepto^{R}	In-fusion cloning	PCR using pGMCS- P_{pacL1}-*pacL1*,GFP11-*ctpC*(MBD)-GFP(1-10) as template with Rv3269(E71A) subst Left/Rv3269(E71A) subst Right primers and circularization by In-fusion reaction	This work
pGMCS-P_{pacL1}-*pacL1*(3EA),GFP11-*ctpC*(MBD)-GFP(1-10)	pDE43-MCS Strepto^{R}	In-fusion cloning	PCR using pGMCS- P_{pacL1}-*pacL1*($E^{71}A$), GFP11-*ctpC*(MBD)-GFP(1-10) as template with subs EA 55-59 Left/subs EA 55-59 Right primers and circularization by In-fusion reaction	This work

Supplementary Table 3. Primers used for plasmid constructions.

Name	Sequence 5'-> 3'
3269 Cter Flag-Am-ctpC-Right	GCACGACCACGACCACGACTACAAGGACGACGA TGACAAATGAGCGCCTCGCCATGACC
3269 gfp11 L2 Fw	CACGACCACGACCACCTGGAAGGATCCGGTGGCGGTG GTTCAGGTGGAGGGTCTGAAAAGCGTGACCACATGG
3269 gfp11 Rv	GTCATGGCGAGGCGCTCAAGATGCATCTGTAATCCCAG
ctpC am left2	CATGGCGAGGCGCTCAAG
ctpC am right	ACCCTGGAAGTGGTATCGG
ctpC Cter His-Av-attB3-Right	TACCGCCTGGACCGCCACCACCACCACCACCACT GAGATAATTCACTGGCCGTCG
clo-B2-3269-Am	GGGGACAGCTTTCTTGTACAAAGTGGAGCGATACT CGACGATTC
clo-B3-3269-Av	GGGGACAACTTTGTATAATAAAGTTGGCTCAGTGGT CGTGGTCGTGC
clo-B3-ctpC-Av	GGGGACAACTTTGTATAATAAAGTTGGCTAGCGGTC CAGGCGGTAGCGG
Del3269-Cterm3-Right	ACTCCAGCGATCAGCGACCTGCACGACTGAGCCA ACTTTATTATACATAGTTGATAATTCACTGG
Del3269-Cterm7-Right	ACTCCAGCGATCAGCTGAGCCAACTTTATTATAC ATAGTTGATAATTCACTGG
Del3269-Cterm-Left	GCTGATCGCTGGAGTGGGCG
Del3269-Nterm7_26-Right	GCGATACAAGTGTTCTTAAAAAAGGCCGCGGCCAAAG
Del3269-Nterm-Left	GAACACTTGTATCGCCATTGCCTG
FL15 left	GGTCCAGGGCTGTCTGGCCT
GFP1-10 Fw inf ctpC	CTTGAGCGCCTCGCCATGGGTGGCACTAGTAGCAA
GFP1-10 Rv L2 inf ctpc	CCGATACCACTTCCAGGGTAGACCCTCCACCTGAACCACC GCCACCGGATCCTTCCAGGGTACCCTTTTCGTTGGGAT
Inf-0968-Am	AAGGTACAGGCAATGGTGTGGCATGGATTCCTAG
Inf-0968-Av	TGGTCGTGGTCGTGCTCAGTGGTCATGACCGTCG
Inf-1993-Am	AAGGTACAGGCAATGGTTACGCATGAGCTATTGG
Inf-1993-Av	TGGTCGTGGTCGTGCTCACTCGTCGACCCTGGCGCCAG
Inf-1993MBM-Av	TGGTCGTGGTCGTGCAGGTCGGCGCCAGCGGGCAGGGGCG
Inf-3269C7-right	GACCTGCACGACCACGACCAC
Inf-3269Del(37-86)-left	GTGGTCGTGCAGGTCAAGCGGCGCTTTGGCCGCGG
Inf-3269Del(53-86)-left	GTGGTCGTGCAGGTCCTTGCGGGTTCCGCGCAGACC
Inf-3269DelC7-left	GTCGTCCTTGTAGTCGCTGATCGCTGGAGTGGGCG
inf-CtpC-MBD-L15	AGGCCAGACAGCCCTGGACCCGCACGCGCGGGGATCAG
InF-ctpC-MutPB-L1	CTCGTTGCAGTGACATTAAGGTCTATATATCGCGAT ATCAATATG
InF-ctpC-MutPB-R1	TGTCACTGCAACGAGCTGCCG
inf CtpC-Nter #1	CTTGAGCGCCTCGCCATGACCCTGGAAGTGGTATCGG
Inf-Flag-right2	GACTACAAGGACGACGATGAC

Inf-P1-left	GAAATGATGTATGCCGTGCTGGTC
Inf-Rv3269-left	CATTGCCTGTACCTTTCTTCC
Inf-Rv3269-right	GCACGACCACGACCACTGAG
infus backbone mVenus Right	CTGGAGGGTTCGGGCGTGAG
infus mCherry Rv	TTTACCTTCCTCGCCTTACTTGTACAGCTCGTCCA
infus mVenus Rv1488 Rv	CACGCCCGAACCCTCCAGTTGAGTCAACCTGGGGGGC
infus P1 Rv1488 Fw	ATGAGAGGAGGATTCAC
Infus pEN41A-P1_x_mCherry Right	GGTGAATCCTCCTCTCATT
infus P1 Rv1488 Fw (pGMC)	AGAGCCTGCGGCATGCATGCAAGGAGCCGTTGCT
infus Rv1488 Fw	CAAGTAAGCGCCTCGCCGTGCAAGGAGCCGTTGCT
mCherry Fw2 w/o start codon	GTGAGCAAGGGCGAGGA
mT av left	GGCGAGGCGCTTACTTGTAG
mTurquoise-Fw #3	CACGACCACGACCACCTGGAGGGTTCGGGCGTCAGC AAGGGTGAGGAAC
mTurquoise-Rv #3	GTCATGGCGAGGCGCTTACTTGTAGAGTTCGTCCATGC
mVenus-Fw #1	GCTACCGCCTGGACCGCCTgGAGGGTTCgGGcGTGAGC AAGGGCGAGGAGC
mVenus-Rv #1	CGGCCAGTGAATTATCGGGGTTACTTGTACAGCTCGT CCATGCCG
pGMC-12-left	GGCGAGGAAGGTAAACAGG
pGMC-12-right	CATGCCGCAGGCTCTCTTTG
pGMC 3269 Am Left	CATTGCCTGTACCTTTCTTCC
pGMC-3269-FLUO-ctpC Left	TCCTCGCCCTTGCTCACCCCCGAACCCT
pGMC-Am-ctpC Right #3	GCGCCTCGCCATGACC
pGMC-Av-attB3-Right #1	GATAATTCACTGGCCGTCG
pGMC del3269 Right	AAGGTACAGGCAATGACCCTGGAAGTGGTATCG
pGMC-Cter-ctpC Left #1	GCGGTCCAGGCGGTAGCG
pGMC-Cter-rv3269 Left #3	GTGGTCGTGGTCGTGC
Rv0968 XFP Fw	CAAGTAAGCGCCTCGCCAGGAGCATCCGCGATGGT
Rv0968 XFP Rv	TCCTCGCCCTTGCTCACGCCCGAACCCTCCAGGTGGT CATGACCGTCGTCCG
Rv1993c XFP Fw	CAAGTAAGCGCCTCGCCAGGAGAACTGACCGTGGT
Rv1993c XFP Rv	CTCCTCGCCCTTGCTCACGCCCGAACCCTCCAGCTCG TCGACCCTGGCGCC
Rv3269(E71A) subst Left	GGCCATCACGTCGGCCA
Rv3269(E71A) subst Right	CCGACGTGATGGCCGCCGCTCGTGAGCGCATC
Rv3269-Flag-Rev	ATCGGCGGGAGAATCGCGCTCATTTGTCATCGTCG

Rv3269-mT-Rev	ATCGGCGGGAGAATCGGCGAGGCGCTTACTTGTAG
Rv3269-P1-Fus-Fw	CAGCACGGCATACATCATTTCGGAAGAAAGGTACAGGCAATGG
SolPacL1Δ3-Fw	GACCTGCACGACTAGGACCACTGAAAGCTTGGC
SolPacL1Δ3-Rv	GCCAAGCTTTCAGTGGTCCTAGTCGTGCAGGTC
SolPacL1Rv	CCAGGGTCATGGCGAAGCTTTCAGTGGTCGTGGTCGTGC
SolPacL1Fw	CCGCCTACGAGATCTTAACCATGGCCGCGGCCAAAGCG
subs EA 55 Right	GAACCCGCAAGGCCGTGGAAGCCGCGGAATCGG
subs EA 55-59 Left	CGGCCTTGCGGGTTCCGCG
subs EA 55-59 Right	GAACCCGCAAGGCCGTGGAAGCCGCGGccTCGGCCCG CCTAAAGG
subs EA 59 Left	CCGCGGCTTCCTCGGCCTTG
subs EA 59 Right	CCGAGGAAGCCGCGGCCTCGGCCCGCCTAAAGG
Tet-P1-Fus-Fw	CGATCCAATATTACGAGATCGGATCGTCGGCACCGTCAC
TetR-Am-Fus-P1	GATCTCGTAATATTGGATCG
TetR-Av-Rv	GGAGCGATACTCGACGTTCTCGGCTCGATGATCCC

Supplementary Table 4. Primers used for RT-qPCR experiments.

Gene	Name	Sequence 5'-> 3'
pacL1	RT 3269 Fw	TGGCGATACAAGTGTTCTTGGCG
	RT 3269 Rv	ACGTCGGCCACCTTTAGGCGG
pacL1 (N-term)	RT 3269Nt Fw1	CAAGTGTTCTTGGCGAAGG
	RT 3269Nt Rv1	TAAGATCTCGTAGGCGGTCA
ctpC	RT ctpC Fw	TCACCATTTTCACCGGGTAT
	RT ctpC Rv	GATGTTGAGCAACCACAGGA
sigA	RT sigA_2F	AAGACACCGACCTGGAACTC
	RT sigA_2R	CGGCATCAGCTTCTTCTTC
rpoB	RT rpoB Fw	TCGTTCTCTGACCCTCGTTTC
	RT rpoB Rv	ACGTGCCCTTCTCGGTCATCA

Supplementary references

1 Cole, S. T. *et al.* Deciphering the biology of *Mycobacterium tuberculosis* from the complete genome sequence. *Nature* **393**, 537-544 (1998).

2 Corpet, F. Multiple sequence alignment with hierarchical clustering. *Nucleic Acids Res.* **16**, 10881-10890 (1988).

3 Cavet, J. S., Graham, A. I., Meng, W. & Robinson, N. J. A cadmium-lead-sensing ArsR-SmtB repressor with novel sensory sites. Complementary metal discrimination by NmtR AND CmtR in a common cytosol. *J. Biol. Chem.* **278**, 44560-44566 (2003).

4 Liu, T. *et al.* CsoR is a novel *Mycobacterium tuberculosis* copper-sensing transcriptional regulator. *Nat. Chem. Biol.* **3**, 60-68 (2007).

5 Notredame, C., Higgins, D. G. & Heringa, J. T-Coffee: A novel method for fast and accurate multiple sequence alignment. *J. Mol. Biol.* **302**, 205-217 (2000).

6 Wishart, D. S., Sykes, B. D. & Richards, F. M. The chemical shift index: a fast and simple method for the assignment of protein secondary structure through NMR spectroscopy. *Biochemistry* **31**, 1647-1651 (1992).

7 Oates, M. E. *et al.* D(2)P(2): database of disordered protein predictions. *Nucleic Acids Res.* **41** (2013).

8 Dereeper, A. *et al.* Phylogeny.fr: robust phylogenetic analysis for the non-specialist. *Nucleic Acids Res.* **36**, W465-469 (2008).

9 Dobson, L., Remenyi, I. & Tusnady, G. E. CCTOP: a Consensus Constrained TOPology prediction web server. *Nucleic Acids Res.* **43**, W408-412 (2015).

10 Arguello, J. M. Identification of ion-selectivity determinants in heavy-metal transport P1B-type ATPases. *J. Membr. Biol.* **195**, 93-108 (2003).

11 Nies, D. H. Efflux-mediated heavy metal resistance in prokaryotes. *FEMS Microbiol. Rev.* **27**, 313-339 (2003).

12 Smith, A. T., Smith, K. P. & Rosenzweig, A. C. Diversity of the metal-transporting P1B-type ATPases. *J. Biol. Inorg. Chem.* **19**, 947-960 (2014).

13 van Kessel, J. C. & Hatfull, G. F. Recombineering in *Mycobacterium tuberculosis*. *Nat. Methods* **4**, 147-152 (2007).

14 Schnappinger, D., O'Brien, K. M. & Ehrt, S. Construction of conditional knockdown mutants in mycobacteria. *Methods Mol. Biol.* **1285**, 151-175 (2015).

15 Ariyachaokun, K., Grabowska, A. D., Gutierrez, C. & Neyrolles, O. Multi-stress induction of the *Mycobacterium tuberculosis* MbcTA bactericidal toxin-antitoxin system. *Toxins* **12**, 329 (2020).

16 Diaz, R., Rech, J. & Bouet, J. Y. Imaging centromere-based incompatibilities: Insights into the mechanism of incompatibility mediated by low-copy number plasmids. *Plasmid* **80**, 54-62 (2015).

17 Guilhas, B. *et al.* ATP-driven separation of liquid phase condensates in bacteria. *Mol. Cell* **79**, 293-303 e294 (2020).

18 Cabantous, S. *et al.* A new protein-protein interaction sensor based on tripartite split-GFP association. *Sci. Rep.* **3**, 2854 (2013).
