# Supplementary material for: Mycobacterial resistance to zinc poisoning requires assembly of P-ATPase-containing membrane metal efflux platforms
Source: Nat Commun. 2022 Aug 12;13:4731. doi: 10.1038/s41467-022-32085-7 (PMC9374683; doi:10.1038/s41467-022-32085-7)
Supplement: Supplementary file 7 — Description of Additional Supplementary Files [file 41467_2022_32085_MOESM7_ESM.pdf]

**Title: Supplementary Data 1**.
**Description:** Occurrence of DUF1490 and DUF6110 proteins in prokaryotes.

**Title: Supplementary Movie 1**.
**Description:** *M. smegmatis* expressing mTurquoise-tagged PacL1 and CtpC under the control of the $P_{pacL1}$(ΔTM) promoter were grown in complete 7H9 medium and live cells were examined using an Eclipse TI-E/B wide field epifluorescence microscope by time lapse microscopy. The movie shows 13 frames with an exposure time of 200 ms and a frame interval of 10 s.

**Title: Supplementary Movie 2.**
**Description:** *M. smegmatis* expressing PacL1 and mVenus-tagged CtpC under the control of the $P_{pacL1}$ promoter were grown in complete 7H9 medium and live cells were examined using an Eclipse TI-E/B wide field epifluorescence microscope by time lapse microscopy. The movie shows 13 frames with an exposure time of 500 ms and a frame interval of 10 s.

**Title: Supplementary Movie 3.**
**Description:** *M. smegmatis* expressing mVenus-tagged Rv1488 under the control of the $P_{pacL1}$ promoter were grown in complete 7H9 medium and live cells were examined using an Eclipse TI-E/B wide field epifluorescence microscope by time lapse microscopy. The movie shows 13 frames with an exposure time of 500 ms and a frame interval of 10 s.
